# Peptide vaccine-conjugated mesoporous carriers synergize with immunogenic cell death and PD-L1 blockade for amplified immunotherapy of metastatic spinal

**DOI:** 10.1186/s12951-021-00975-5

**Published:** 2021-08-12

**Authors:** Zhenqing Wang, Liang Chen, Yiqun Ma, Xilei Li, Annan Hu, Huiren Wang, Wenxing Wang, Xiaomin Li, Bo Tian, Jian Dong

**Affiliations:** 1 Department of Orthopaedic Surgery, Zhongshan Hospital, Fudan University, Shanghai, 200032 People’s Republic of China; 2 Cancer Center, Zhongshan Hospital, Fudan University, Shanghai, 200032 People’s Republic of China; 3 Materdicine Lab, School of Life Sciences, Shanghai University, Shanghai, 200444 People’s Republic of China; 4 Department of Orthopaedic Surgery, Shanghai Baoshan District Wusong Center Hospital, Zhongshan Hospital Wusong Branch, Fudan University, Shanghai, 200940 People’s Republic of China

**Keywords:** Spine metastasis, Peptide vaccine, Immunogenic cell death, Photodynamic therapy, Programmed cell death protein 1/programmed cell death ligand 1 (PD-1/PD-L1) blockades

## Abstract

The clinical treatment of metastatic spinal tumor remains a huge challenge owing to the intrinsic limitations of the existing methods. Programmed cell death protein 1 (PD1)/programmed cell death ligand 1 (PD-L1) pathway blockade has been explored as a promising immunotherapeutic strategy; however, their inhibition has a low response rate, leading to the minimal cytotoxic T cell infiltration. To ameliorate the immunosuppressive microenvironment of intractable tumor and further boost the efficacy of immunotherapy, we report an all-round mesoporous nanocarrier composed of an upconverting nanoparticle core and a large-pore mesoporous silica shell (UCMS) that is simultaneously loaded with photosensitizer molecules, the IDO-derived peptide vaccine AL-9, and PD-L1 inhibitor. The IDO-derived peptide can be recognized by the dendritic cells and presented to CD8^+^ cytotoxic T cells, thereby enhancing the immune response and promoting the killing of the IDO-expressed tumor cells. Meanwhile, the near-infrared (NIR) activated photodynamic therapy (PDT) could induce immunogenic cell death (ICD), which promotes the effector T-cell infiltration. By combining the PDT-elicited ICD, peptide vaccine and immune checkpoint blockade, the designed UCMS@Pep-aPDL1 successfully potentiated local and systemic antitumor immunity and reduced the progression of metastatic foci, demonstrating a synergistic strategy for cancer immunotherapy.

**Supplementary Information:**

The online version contains supplementary material available at 10.1186/s12951-021-00975-5.

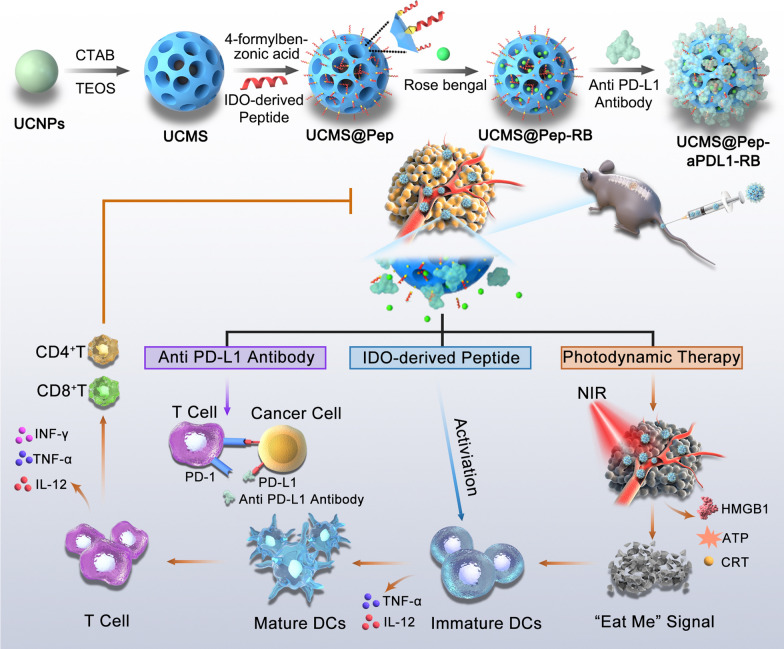

## Introduction

Non-small cell lung cancer (NSCLC) is one of the most common malignant tumors of the lung and has the second highest incidence and highest morbidity worldwide [1]. Although effective treatments can prolong the survival of NSCLC patients, the bone metastasis caused by advanced lung cancer remains an intractable problem [2, 3]. In particularly, spinal metastasis typically causes severe complications, including vertebral pathological fractures, spinal cord suppression, and neurological dysfunction, gravely affecting the quality of life of the patients [4–6]. Current treatment approaches for metastatic spinal tumors are mainly limited to traditional chemotherapy, radiotherapy, and surgery. Surgical treatments often result in infection and tumor recurrence, and are not applicable for multiple metastases [7]. Furthermore, chemotherapy and radiotherapy have inevitable side effects, such as thrombocytopenia, liver and kidney toxicity, radiation myelopathy, and radiculitis [8]. Dose limitation and drug resistance also dramatically impair therapeutic outcomes [9]. Therefore, the development of new strategies to efficiently combat metastatic spinal tumors is a challenging area that must be urgently explored.

Recently, immunotherapy has emerged as a revolutionary technology for cancer treatment owing to the success of immune checkpoint block (ICB) [10]. ICB therapy could potentially reverse an immunosuppressive tumor microenvironment and further induce antitumor immunologic response to eliminate local and disseminated tumors [11]. However, there are several challenges to the wide application of ICB therapy. For instance, the systemic administration of monoclonal antibodies may cause “on-target but off-tumor” effects, resulting in adverse immune-related events, such as dermatitis, colitis, and hepatitis [12]. Moreover, the programmed cell death protein 1 (PD1)/programmed cell death ligand 1 (PD-L1) pathway has a low response rate of approximately 30% in inhibiting lung cancer, as characterized by the minimal cytotoxic T cell infiltration [13–15]. Therefore, a successful immunotherapy platform should potentiate cytotoxic T cell immune responses by modulating an immunosuppressive microenvironment, and significantly enhance the susceptibility of tumors to immunotherapy with reduced off-target toxicity and immune-related adverse effects [16].

Nanomedicine offers unprecedented potential in increasing the safety and therapeutic efficacy of immunotherapies [17–19]. Engineered nanoparticles with various therapeutic effects have been recognized as an attractive solution in promoting antitumor immune response by inducing the immunogenic cell death (ICD) of tumor cells, which involves the release of damage-associated molecular patterns (DAMPs), including adenosine triphosphate (ATP), high-mobility group protein B1 (HMGB1), and calreticulin (CRT) [20]. Thus, the combination of nanomedicine-mediated ICD and ICB therapy has been widely developed to achieve superior therapeutic efficacy and robust antitumor immunity [21, 22]. Additionally, cancer vaccines have also been considered as a strategy to activate tumor-specific T cell immunity. Previous clinical studies confirmed that a tumor antigen-derived peptide vaccine can induce T cell activation and promote the infiltration of CD4^+^ and CD8^+^ T cells to kill antigen-expressing tumor cells [23, 24]. In addition, several studies have demonstrated improved antitumor efficacy by incorporating peptide-based vaccines with PD-1/PD-L1 blockade, compared to in vitro and in vivo PD-L1 inhibitor or peptide alone [25]. However, the non-specific binding with normal tissues and rapid in vivo clearance of cancer vaccines should be carefully established. In this regard, it is imperative to develop a multifunctional nanoplatform that integrates nanomedicine-mediated ICD, cancer vaccination, and ICB therapy to achieve satisfactory antitumor efficacy against advanced or metastatic tumors with minimal immune-related adverse effects [26].

Herein, monodispersed dendritic mesoporous silica-coated upconverting nanoparticles (UCMS) were synthesized and utilized as a versatile nanoplatform for photodynamic therapy (PDT)-mediated ICD and peptide vaccine-potentiated ICB immunotherapy. An orthotopic metastatic spinal tumor model was established to evaluate its efficacy in advanced cancer for the first time. The large pores of the dendritic mesoporous silica allow efficient loading of photosensitizer molecules and the PD-L1 antibody, atezolizumab. UCMS facilitate near-infrared (NIR)-triggered PDT, inducing the ICD of tumor cells, and subsequently recruit and activate immune cells for immunotherapy. Moreover, a peptide vaccine (AL-9) derived from indoleamine 2,3-dioxygenase (IDO) was conjugated on the surface of the mesoporous silica and applied as the neoantigen to elicit neoantigen-specific T cell response. By combining NIR laser-mediated PDT, peptide-augmented immune response, and ICB therapy, a strong synergistic and potent antitumor efficacy was achieved on the metastatic spinal tumors.

## Methods

### Synthesis of UCMS

First, the SiO_2_ coated upconverting nanoparticles (UCNPs) were synthesized by reversed microemulsion method. 1.0 mL of NaYF_4_: Yb/Er@NaYF_4_ solution was added into 30 mL of cyclohexane containing 8.4 mL butanol and 15 mL of TritonX-100. Then 300 µL of ammonium hydroxide solution was slowly added and the mixture was stirred for 0.5 h, followed by the addition of 200 µL of tetraethyl orthosilicate (TEOS). After further reacting overnight, the SiO_2_ coated UCNPs was obtained by centrifugation and washed with anhydrous ethanol.

Next, the above SiO_2_ coated UCNPs were dispersed in 30 mL of ultrapure water containing 1.5 g CTAB and 0.15 mL of triethanolamine under 60 °C. Then an upper solution of 10.0 mL cyclohexane and 100 µL of TEOS was added. The reaction was maintained for 24 h under 60 °C. The UCMS was collected and washed by centrifugation. Finally, the CTAB was repeatedly extracted by acid-ethanol solution under 80 °C. The modification of amino groups and 4-formylbenzoic acid was consistent with our previous work [27].

### Preparation of UCMS@Pep-aPDL1

To conjugate the peptide onto UCMS, 10 mg of the functionalized UCMS was dispersed in 5.0 mL of dimethyl-sulfoxide. After that, 5.4 mg of N-(3-Dimethylaminopropyl)-3-ethylcarbodiimide hydrochloride (EDC) and 3.8 mg of N-Hydroxy succinimide (NHS) was added and the mixture was stirred under room temperature for 2 h. The AL-9 peptide was further added, the reaction was continued overnight. Then the peptide conjugated UCMS (UCMS@Pep) was obtained by centrifugation.

For the loading of antibody, 10 mg of UCMS@Pep and 10 mg of anti-PDL1 antibody (Atezolizumab) were added into 5.0 mL of water and stirred overnight to obtain the UCMS@Pep-aPDL1. The fluorescein Isothiocyanate (FITC)-labeled aPDL1 was loaded into the UCMS@Pep by the same protocol and all supernatant solution during the loading process was collected to measure the loading content of aPDL1. Similarly, the loading of Rose Bengal (RB) was performed in 10 mL of water containing 10 mg of nanoparticles and 4.0 mg of RB, the excessive molecules were removed by centrifugation and the supernatant solution was collected for the measurement of UV-vis spectrophotometer. The loading efficiency was calculated by the equation: loading efficiency (%) = (the weight of drug in carrier/the weight of drug-loaded carrier) × 100%. To assess the release of RB from UCMS@Pep, the drug-loaded nanoparticles were immersed in 2 ml of phosphate buffer solution (PBS) with corresponding pH value and incubated in a shaker at 37 °C. At specific time point, the dispersions were centrifuged and the supernatant was withdrawn for the measurement of UV-vis spectrophotometer.

### Cells and culture

Tumor-derived mouse cell line, Lewis murine lung carcinoma cells (LLC) were purchased from National Collection of Authenticated Cell Cultures and cultured in Dulbecco’s Modified Eagle Medium (keygentec, China) with 10% fetal bovine serum (FBS, TICO Europe, Netherlands), 100 U/mL penicillin, and 100 µg/mL streptomycin (keygentec, China) at 37 °C in an atmosphere of 5 % CO_2_.

DCs harvest and culture was done as described in previous study [28]. Briefly, tibia and fibula were obtained from 6 to 8 w C57BL/6 mice under aseptic conditions. Bone marrow cells were isolated from bone marrow cavity and cultured in RPMI-1640 medium (keygentec, China) supplemented with 10 % FBS, 100 U/mL penicillin, GM-CSF (30 ng/mL) and IL-4 (20 ng/mL) (PeproTech, USA) for 6 h. Then the suspensive and non-adherent cells were collected and seeded into a 24-well plate at a number of 5 × 10^5^ cells with complete RPMI-1640 medium with GM-CSF (30 ng/mL) and IL4 (20 ng/mL). Then the culture medium was half renewed every 3 days. At day 7 the immature dendritic cells were used for next experiment.

### Cell viability/Cytotoxicity Experiment in vitro

LLC cells (5 × 10^3^ cells) were seeded into a 96-well plate (Nest, China) with 100 µL DMEM medium each well and cultured for 12 h to allow cell adhesion. Then the culture medium was refreshed with blank UCMS, UCMS@Pep, and UCMS@Pep-RB at difference concentrations (0, 3.125, 6.25, 12.5, 25, 50, 100, 200 µg/mL). After 6 h, these cells were irradiated with or without 980 nm laser (10 min, 1.0 W/cm^2^). Next, the cells were cultured for another 24 h and subsequently exposed to CCK-8 work solution (90 µL DMEM + 10 µL CCK-8 stock solution), then cultured with another 30 min at 37 °C. The plate was gently shaking for 1 min, and the absorbance of each well was detected at the wavelength of 450 nm using microplate reader. Cell viability was determined according to the instructions.

### Dead or live cell staining experiment

LLC cells were seeded into a 48-well plate (Nest, China) at a density of 4 × 10^4^ cells per well and incubated for 12 h. Different interventions were conducted for LLC cells: Control, NIR laser, UCMS@Pep, UCMS@Pep-RB + NIR laser. After 6 h incubation, NIR laser and UCMS@Pep-RB + NIR laser groups were irradiated by 980 nm laser (1.0 W/cm^2^) for 10 min. After incubated for another 24 h, the live cells were stained by Calcein AM (λ_ex_ = 490 nm, λ_em_ = 515 nm) and dead cells are stained with pyridine iodide (PI) (λ_ex_ = 535 nm, λ_em_ = 617 nm) for 30 min, and directly imaged by using an inverted fluorescence microscope.

### Cell apoptosis analysis in vitro

LLC cells (4 × 10^4^ cells/well) were seeded into a 48-well plate with 500 µL DMEM medium and cultured for 12 h. Then cells were divided into different groups as follows: Control, NIR laser, UCMS@Pep, UCMS@Pep-RB + NIR (100 µg/mL). After incubated for 6 h, NIR laser and UCMS@Pep-RB + NIR laser groups were irradiated by 980 nm laser (1.0 W/cm^2^) for 10 min. After incubated for another 24 h, these cells were digested and collected, washed with PBS twice and resuspended. Then propidium iodide and FITC-labeled Annexin V (Beyotime Biotechnology, China) was added to the suspended cells for staining accoding to the manufacturer’s instruction. Finally, the cells were analyzed by flow cytometry.

### Intracellular ROS measurement

For the detection of ROS, LLC cells (4 × 10^4^ cells/well) were seeded into 48-well plates with 500 µL DMEM medium and then cultured for 12 h. After adhere completely, cells were incubated with UCMS@Pep or UCMS@Pep-RB at the concentration of 100 µg/mL. After 6 h incubation, the cells were illuminated with or without NIR laser (10 min, 1.0 W/cm^2^) and then stained with DCFH-DA (Beyotime Biotechnology, China) for 30 min. After that, the cells were washed with PBS for three times and observed using an inverted fluorescence microscope at an excitation wavelength of 488 nm.

### Detection of essential ICD biomarkers in vitro

Cell surface expression of CRT and release of HMGB1 was detected by immunofluorescence. For CLSM observation, LLC cells were seeded into 24-well plates (2 × 10^4^ cells/well), and cultured with 1.0 mL DMEM medium for 12 h. These cells were treated as follow: untreated as control group, NIR laser, UCMS@Pep, UCMS@Pep-RB + NIR laser at an equal amount of nanoparticle (100 µg/mL) for 6 h incubation. Then the NIR laser irradiation was performed for the groups of NIR laser and UCMS@Pep-RB + NIR laser, followed by another 12 h incubatioin. Thereafter, the cells were fixed with paraformaldehyde for 15 min, blocked by 2 % BSA buffer for 1 h, and incubated with anti-calreticulin antibody (Proteintech, China) or anti-HMGB1 antibody (Proteintech, China) overnight at 4 °C. Further, the cells were labeled with Alexa Fluor 488-conjugated secondary antibody for 1 h at room temperature. Next the cells were stained with phalloidin (5 µg/mL) for 1 h, and further stained with DAPI (300 nM) for 15 min. Last, the images were captured by CLSM at excitation wavelengths of 488 nm for CRT or HMGB1, 593 nm for phalloidin and 405 nm for DAPI.

### ELISA kit for ATP and HMGB1 in vitro

Extracellularly released ATP and HMGB1 were examined via ELISA Assay. The cell supernatant of different groups mentioned above was collected. The level of ATP and HMGB1 was detected by the ELISA kit in accordance with the manufacturer’s protocol. Briefly, 50 µL of standard or samples were added to the 96-well plate. The standard wells and sample wells were all mixed with 100 µL of enzyme-conjugate except the blank well, covered with an adhesive strip and incubated for 60 min at 37 °C. Then the samples were washed with PBS for 4 times. Afterwards, 50 µL of substrate A and 50 µL of substrate B were added to each well, gently mixed and incubated for 15 min at 37 °C. Then 50 µL of stop solution was added to each well. The optical density (O.D) at 450 nm was detected using a microtiter plate reader within 15 min.

### In vitro dendritic cell maturation

The immature DCs harvested as described above were seeded into the lower chamber of a 24-well plate. Lewis lung carcinoma (LLC) cells were planted into the upper chamber of the transwell plate. After the cells were confluent, free UCMS, UCMS@Pep, and UCMS@Pep-RB were added. After irradiated with or without NIR laser, the upper chambers of the transwell plate were inserted into the lower chamber with immature DCs and incubated for another 24 h. Then the immature DCs were collected, stained with CD11c, CD80, and CD86, and then analyzed by flow cytometry to detect mature CD11c^+^CD80^+^CD86^+^ DCs. The supernatants of the DCs medium under different treatments were collected and used for further ELISA tests to detect the level of IL-12 and TNF-α. The procedure was performed with the same protocol as above Elisa experiment.

### Animal

Female C57/BL6 mice (6–8 weeks old) were purchased from Animal experiment center of Zhongshan Hospital, Fudan University (Shanghai, China). All animal procedures met the requirements of the Animal Ethics Committee of Shanghai Zhongshan Hospital, Fudan University (2019 -144). All the mice were reared in Specific Pathogen Free (SPF) room.

### Spinal metastasis mouse model

Spinal metastasis mouse model was used in experiments in vivo. After euthanization by intraperitoneal injection of 1% pentobarbital sodium at a dose of 6 µL/g body weight, LLC-luc cells (5 × 10^5^ cells/25µL) were injected into the fourth or fifth lumbar vertebral body via an intermuscular approach at left decubitus. At 7 days after injection, the In Vivo Imaging System (IVIS) was used to detect tumor formation.

### Anti-tumor effect evaluation

Spinal tumor-bearing mice were randomly divided into five groups: (1) PBS, (2) aPDL1: Atezolizumab, (3) UCMS@Pep, (4) UCMS@Pep-aPDL1, (5) UCMS@Pep-aPDL1-RB + NIR laser (1.0 W/cm^2^, 10 min). All groups were intravenously injected with the same volume of sample dispersions (200 µL, 4 mg/mL) every seven days, three times in total. Correspondingly, the PDT was implemented at 12 h post-injection. The body weight and tumor size of each mouse were documented. The progression of tumors was evaluated by an in vivo imaging system (IVIS) every seven days. After IVIS at each point, three mice in every group were euthanized, and their tumors were harvested to weigh and measure the length (L, mm) and width (W, mm), and the volume was calculated according to the formula: Volume (mm^3^) = 0.52 × L × W^2^.

### Examination of DCs, CD4^+^ and CD8^+^ T lymphocytes in vivo

To study the immunological response induced by UCMS@Pep-aPDL1, tumor tissues and spleens of mice were collected in every treatment group after different treatments. Tumor tissues and spleen tissues were cut into small pieces and put into a glass homogenizer containing PBS (pH 7.4) with 2 % heat-inactivated fetal bovine serum. Then, the single-cell suspension was prepared by gentle pressure with a homogenizer without the addition of digestive enzymes. Subsequently, the cells were washed with PBS (pH 7.4) and filtrated with a 200-mesh cell sieve. The remaining cells were centrifugated for 10 min at 1000 rpm twice. Then, the cells were diluted to 1 × 10^6^/100 µL and stained with CD3, CD4, CD8, and CD25 antibodies, Foxp3 for CD4^+^, CD8^+^, Treg cell, and CD11c, CD80, and CD86 antibodies for mature DCs according to the manufacturer’s protocols. Finally, flow cytometry analysis was performed according to the manufacturer’s instructions.

### Cytokine detection and immunofluorescence assay in vivo

The blood samples were harvested by removing the eyeball. Then serum samples were isolated for further analysis. TNF-α, IFN-γ and IL-12 were analyzed with ELISA kits (Kenuodi, China), according to the protocols. TNF-α, IFN-γ and IL-12 were analyzed with ELISA kits (Kenuodi, China), according to the protocols. Tumor sections were analyzed with immunofluorescence staining for CD4^+^ and CD8^+^ T cell, TNF-α, IL-12.

### Statistical analysis

One or two-way ANOVA followed by Bonferroni’s post-test was applied to assess the statistical significance of differences between multiple treatment groups. Data were analyzed using GraphPad Prism 8.0 (GraphPad Software).

## Results and discussion

### Synthesis and characterization of UCMS@Pep-aPDL1

**Fig. 1:**
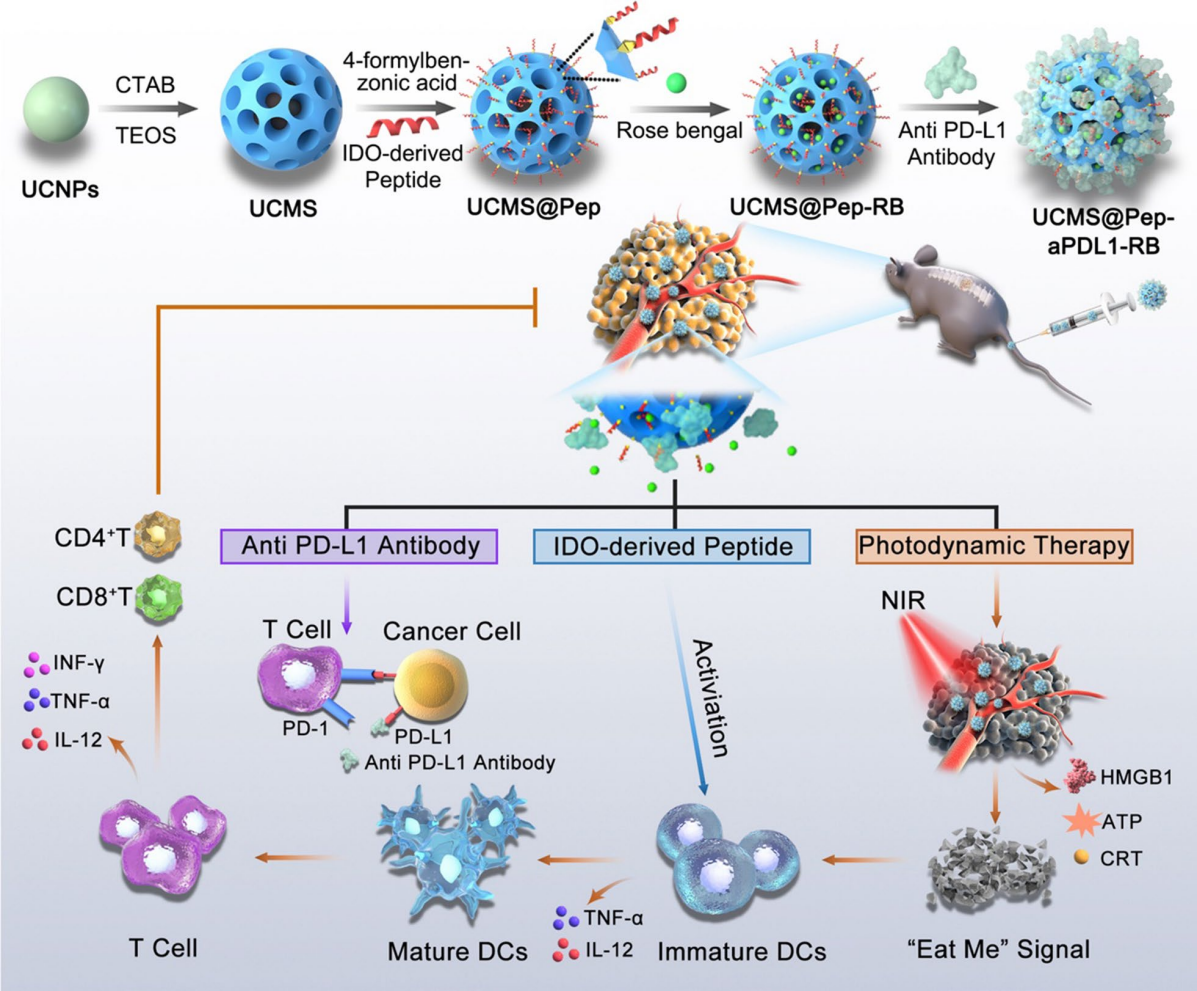
Schematic illustration of the preparation of functional nanocarrier for photodynamic and peptide vaccine-amplified immunotherapy of spinal tumor

**Fig. 2:**
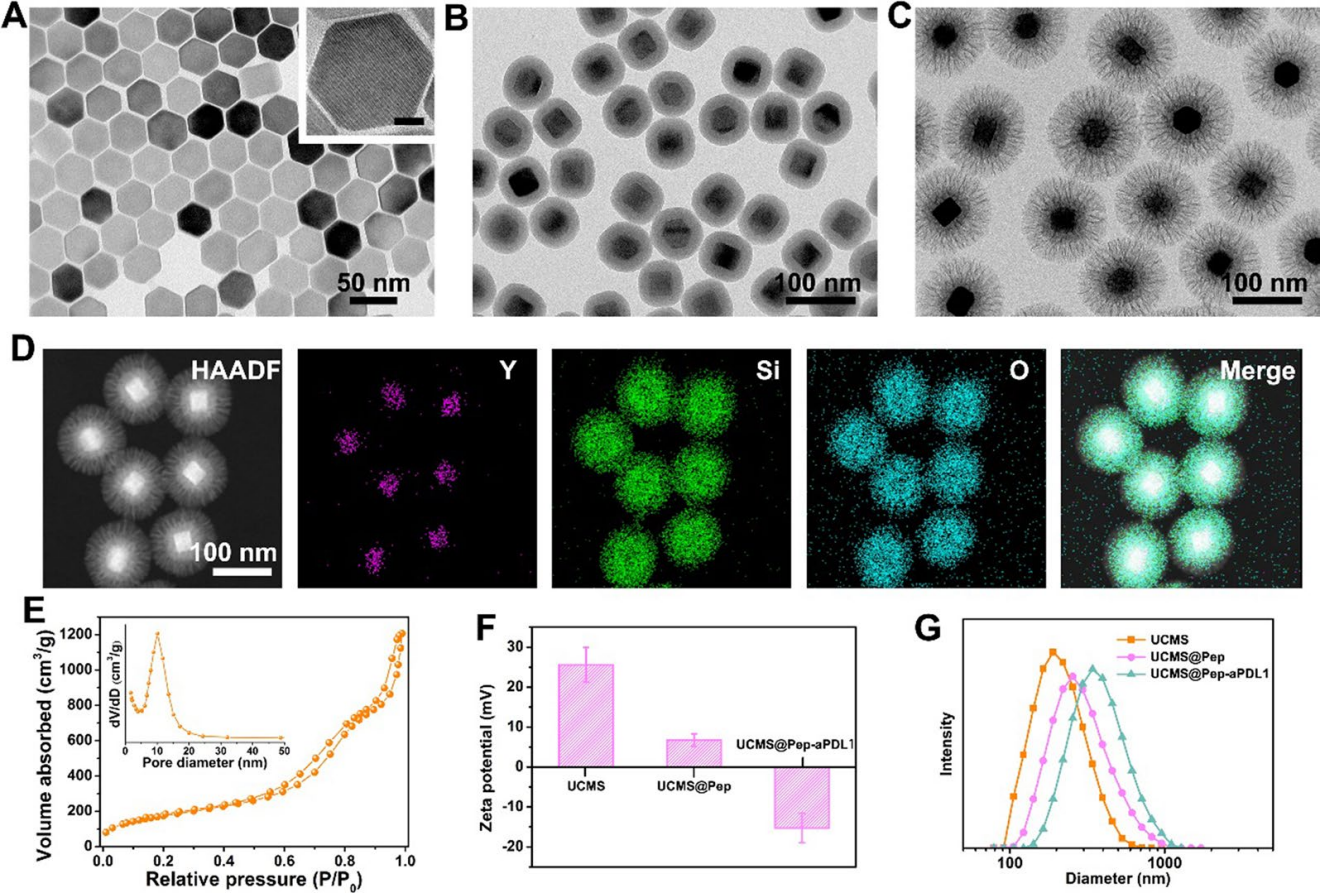
TEM images of (**A**) UCNPs (inset shows the corresponding high-magnification image; scale bar = 10 nm), **B** UCNPs@SiO_2_, and (**C**) UCMS. **D** High-angle annular dark field (HAADF) scanning TEM image and element mapping of the prepared UCMS. **E** Nitrogen adsorption isotherm of the prepared UCMS. **F** Zeta potential and (**G**) size distributions of UCMS, UCMS@Pep, and UCMS@Pep-aPDL1

The preparation of UCMS@Pep-aPDL1 is illustrated in Fig. 1. Upconverting nanoparticles (UCNPs, NaYF_4_:Yb/Er@NaYF_4_) were first synthesized by a thermal decomposition method and successively coated with a layer of dense SiO_2_ and large-pore mesoporous silica by sol-gel method. The transmission electron microscopy (TEM) images show the hexagonal morphology of the prepared UCNPs with an average diameter of approximately 40 nm (Fig. 2A). After coating with silica shell, the uniform and monodispersed morphology was maintained with no abnormal aggregation (Fig. 2B–D and Additional file 1: Fig. S1). The dendritic large pores of the mesoporous silica shell can be clearly observed in the TEM image. The Brunauer–Emmet–Teller surface area and pore volume of UCMS are approximately 652 m^2^/g and 1.88 cm^3^/g, respectively (Fig. 2E). Moreover, UCMS exhibits broad pore size distributions with a strong peak at approximately 11 nm, which facilitates the efficient storage of peptides and antibodies with large molecule sizes.

The prepared UCMS was applied as a vehicle to carry immunotherapy-related cargos. First, the UCMS was functionalized with 3-aminopropyltriethoxysilane and 4-formylbenzoic acid to form an acid–labile benzoic–imine linker to conjugate peptide AL-9 (Additional file 1: Figs. S2 and S3). The Fourier transform infrared spectra demonstrate the immediate anchorage of peptide onto the UCMS surface (Fig. S4). Meanwhile, the PD-L1 antibody atezolizumab is efficiently loaded into the large pores of the UCMS, and the loading efficiency was determined to be 7.4% (Additional file 1: Fig. S5). The resultant UCMS@Pep and UCMS@Pep-aPDL1 have negative surface potentials (Fig. 2F) with mean diameters that are larger than that of free UCMS, confirming the superior loading capacity of the resulting UCMS. Additional file 1: Fig. S6 has shown the cellular uptake of UCMS@Pep-RB in vitro.

### Photon Upconversion and therapeutic effect of in vitro of UCMS@Pep

**Fig. 3:**
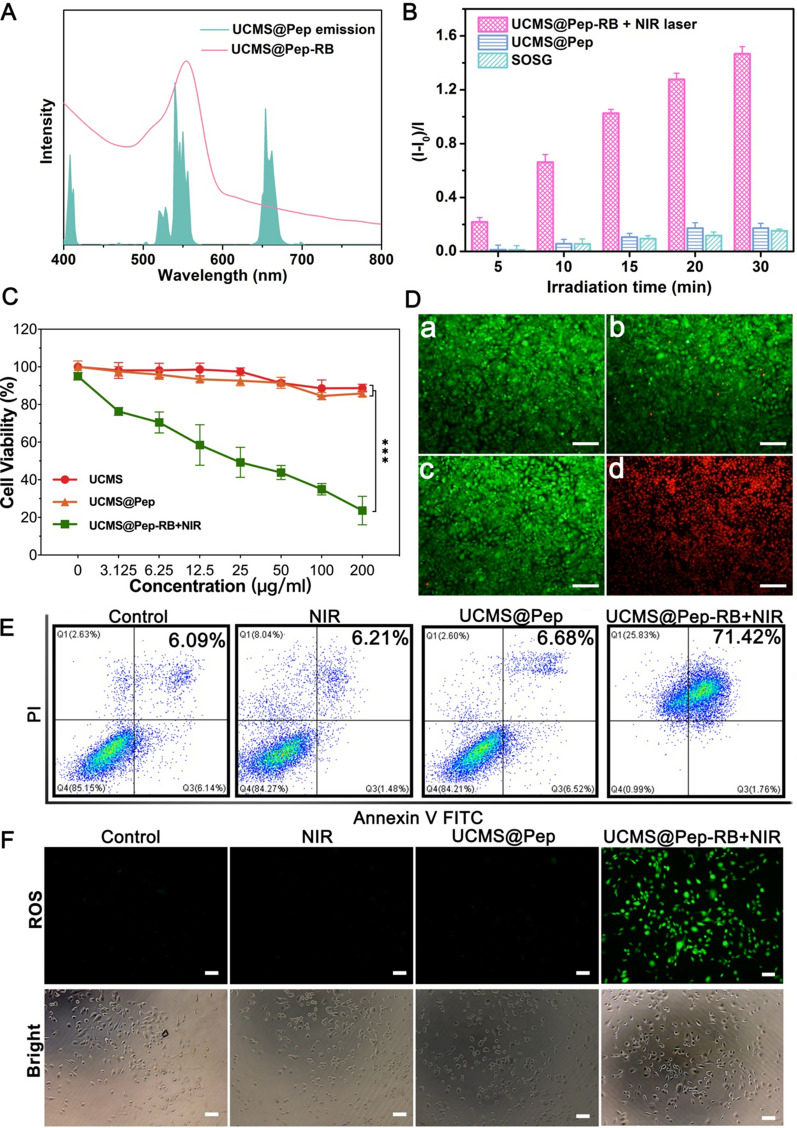
In vitro photon conversion and cellular cytotoxicity of UCMS@Pep. **A** UV-vis spectrum of UCMS@Pep-RB (red line) and up-conversion emission spectrum of UCMS@Pep under excitation at 980 nm (green color). **B** Effects of irradiation time on the fluorescence intensity change of SOSG at 525 nm for different samples. **C** Relative viability of LLC cells incubated with different concentrations of blank nanoparticles, UCMS@Pep alone, and UCMS@Pep with NIR laser. **D** Results of calcein-AM & PI staining that was used to discriminate between live and dead cells (a: Control, b: NIR laser, c: UCMS@Pep, d: UCMS@Pep-RB + NIR laser, scale bar = 100 μm). (**E** Flow cytogram showing the results of the apoptosis assay based on the annexin V-FITC and propidium iodide (PI) staining of LLC cells after different treatments. **F** Corresponding inverted fluorescence microscopy images of DCFH-DA probe–stained LLC cells used to evaluate the overall intracellular ROS generation (scale bar = 50 μm). (*P < 0.05, **P < 0.01, ***P < 0.001, n = 3)

In addition to loading the immune stimulator, the photon upconversion of UCNPs can be used to implement NIR laser-activated PDT. The upconversion luminescence spectrum of UCMS exhibits major emission peaks at 523, 541, and 655 nm (Fig. 3A). Rose Bengal (RB) was selected and loaded into UCMS@Pep-aPDL1 owing to the efficient Förster resonance energy transfer between RB and UCNPs, as demonstrated in our previous report [27]. The loading efficiency of RB was calculated to be 13.59 %, implying that the large pores of the mesoporous silica allow a high loading efficiency of photosensitizers [29]. The faster release of RB under acidic tumor microenvironment is also confirmed in vitro (Additional file 1: Fig. S7), which can ensure the efficient photodynamic effect of RB-loaded nanocomposite upon the specific NIR laser excitation. The photodynamic effect of the RB-loaded UCMS@Pep (denoted as UCMS@Pep-RB) was evaluated using singlet oxygen sensor green (SOSG). Accordingly, the fluorescent emission of SOSG significantly increases with the stimulation of UCMS@Pep-RB and NIR laser, while it remains almost constant for UCMS@Pep-RB or NIR laser alone (Fig. 3B). This suggests the efficient generation of reactive oxygen species (ROS) under the illumination of 980 nm NIR laser. Therefore, the remarkable NIR-activated PDT of UCMS@Pep-RB is expected to induce the ICD of tumor cells and boost the synergistic effect of the designed photo-immunotherapy.

To evaluate the intracellular cytotoxicity of UCMS@Pep, the cell counting Kit-8 (CCK-8) assay was employed. As illustrated in Fig. 3C, the cell viability did not exhibit cytotoxicity against the LLC cells even at a high UCMS concentration of 200 µg/mL. In addition, cytotoxicity of UCMS@Pep to LLC cells is very low. Meanwhile, UCMS@Pep-RB can efficiently kill LLC cells under NIR irradiation based on its concentration. This can be ascribed to the effective cell uptake and NIR laser-activated photodynamic effect of UCMS@Pep-RB.

Subsequently, the live and dead assay was performed to further study the killing effect of UCMS@Pep-RB. Figure 3D shows the nearly non-existent dead cells in UCMS@Pep, control, and NIR laser groups. In contrast, UCMS@Pep-RB + NIR laser group exhibits the strongest red fluorescence intensity from the dead cells stained with propidium iodide (PI), indicating the remarkable cancer cell killing efficacy of NIR-activated PDT. Annexin V-FITC and PI staining assays were used to evaluate the therapeutic efficacy of UCMS@Pep-RB through flow cytometry. Figure 3E displays the ability of UCMS@Pep-RB + NIR laser to induce dramatic apoptosis of LLC cells that are significantly higher than those of NIR laser and UCMS@Pep alone.

Given the fact that the UCMS@Pep-RB can be efficiently internalized into the LLC cells and exert photodynamic efficacy under NIR laser, the intracellular generation of ROS was investigated. ROS is a family of molecules composed of free oxygen characterized by a short life and high responsiveness. Furthermore, ROS is involved in motivating the danger-signaling pathway that promotes the presentation of DAMPs to immune cells in a tumor microenvironment [30]. Here, 2,7-dichlorodihydrofluorescein diacetate (DCFH-DA), which is a typical probe that can be oxidized by ROS to emit green fluorescence, was used to determine intracellular ROS generation. As shown in Fig. 3F, there is nearly no green fluorescence observed in the LLC cells treated with NIR laser or UCMS@Pep alone, while UCMS@Pep-RB with NIR laser exhibits a bright green fluorescence owing to the excellent ROS generation capacity, thereby inducing the death of cancer cells.

### Expression of ICD markers and DCs maturation for enhanced ICD for in vitro UCMS@Pep with NIR laser

As noted in Sect. "Photon Upconversion and therapeutic effect of in vitro of UCMS@Pep", UCMS@Pep-RB with NIR laser could promote apoptosis and necrosis of LLC cells. Apoptotic or necrotic cancer cells can express or release DAMPs that generate an “eat me” signal to elicit immune system, recruit immune cells [31], and subsequently promote ICD, which have a central effect in immunotherapy [32, 33]. CRT, HMGB1, and ATP are noted as key DAMPs [33]. The release of ATP can recruit DCs to the tumor and induce the maturation of bone marrow-derived DCs. At the same time, ATP can also enhance the function of the innate immune system by promoting the proliferation of NK cells. Calreticulin is locating in the endoplasmic reticulum. Photodynamic therapy increases the generation of ROS in tumor cells, which in turn regulates the endoplasmic reticulum stress, promotes the exposure of CRT to the tumor microenvironment, forming the “Eat Me” signal. This signal can recruit dendritic cells and promote its maturity. HMGB1 is a cytokine related to inflammatory response which is locating in the cytoplasm and nucleus. HMGB1 can be exposed on the surface of apoptotic cells after immunogenic cell death, and recruits immature dendritic cells and promotes their maturation by binding to multiple receptors, enhancing functions of immune recognition, antigen processing and presentation [33]. To evaluate whether the NIR-activated photodynamic effect of UCMS@Pep-RB could effectively induce ICD in vitro, we employed immunofluorescence and ELISA kits to detect the DAMPs expression.

**Fig. 4:**
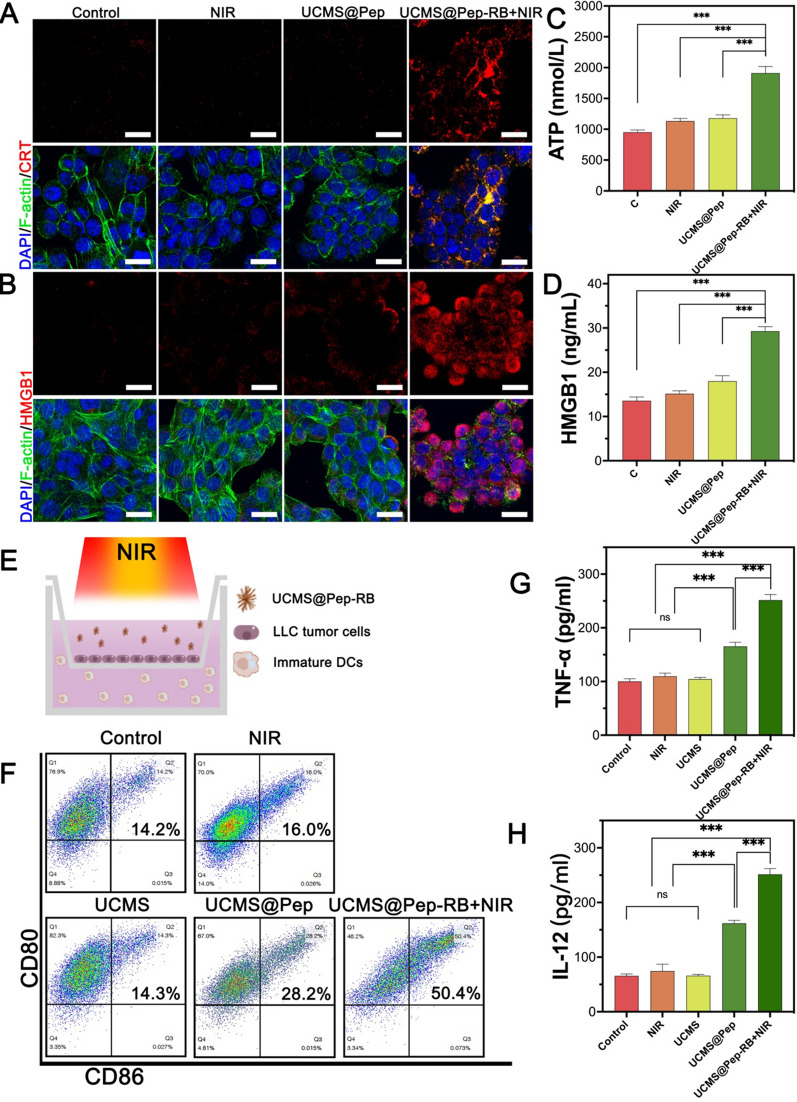
Intracellular ICD marker and DCs maturation of UCMS@Pep in vitro. **A** CRT exposure and (**B**) HMGB1 analysis by immunofluorescence using CLSM (scale bar = 25 μm). Release of (**C**) ATP and (**D**) HMGB1 detected by ELISA kits for different interventions. **E** Schematic of the trans-well DCs maturation system. **F** Flow cytometry results for mature DCs after different treatments. Levels of cytokines (**G**) TNF-α and (**H**) IL-12 in the culture medium. (*P < 0.05, **P < 0.01, ***P < 0.001, *n* = 3)

To evaluate the expression of CRT and release of ATP and HMGB1, the LLC cells were treated with complete culture medium (as control), NIR laser, UCMS@Pep, and UCMS@Pep-RB + NIR laser. The expression of CRT and HMGB1 was evaluated by confocal laser scanning microscopy. The release of ATP and HMGB1 was further evaluated using ELISA kits. Figure 4A, B, Additional file 1: Figs. S8, S9 show the significantly higher expression levels of CRT and HMGB1 for the LLC cells treated with UCMS@Pep-RB + NIR laser as compared to the other groups. Meanwhile, treatment with only NIR irradiation or UCMS@Pep did not promote the levels of CRT and HMGB1. Additionally, the LLC cells in the UCMS@Pep-RB + NIR laser group have a disrupted F-actin (green fluorescence). The results of the ELISA kits illustrate the most prominent release levels of ATP and HMGB1 with the treatment of UCMS@Pep-RB + NIR laser (Fig. 4C and D). Meanwhile, there was no significant difference on the release levels of ATP and HMGB1 for the control and UCMS@Pep. Therefore, UCMS@Pep-RB + NIR laser could prominently induce the release of DAMPs.

Next, the capability of the UCMS@Pep nano-system to promote dendritic cells (DCs) maturation in vitro was evaluated. DCs are among the most powerful antigen-presenting cells that play a vital role in initiating the host immune system [34, 35]. Peptide-based vaccines can be recognized by immune cells, such as DCs, and provide tumor antigens to promote their maturation [36]. AL-9 peptide vaccine was derived from the tumor-associated molecule IDO. Thus, we hypothesized that integrating the peptide vaccine and photodynamic effect of UCMS@Pep-RB could significantly promote the in vitro DCs maturation. The Transwell co-culture system was used to evaluate the potential of UCMS@Pep to promote DCs maturation. As demonstrated in Fig. 4E, LLC cells and DCs were added to the upper and lower chambers of the Transwell system, respectively. LLC cells were then treated with NIR laser, UCMS, UCMS@Pep, or UCMS@Pep-RB + NIR laser. After 24 h of incubation, flow cytometry was used to detect the percentage of CD11c^+^CD80^+^CD86^+^ DCs as mature DCs. As shown in Fig. 4F, there are equal amounts of maturate DCs in the UCMS and control groups, indicating that only tumor cells and free UCMS could not induce DCs maturation. The DCs treated with LLC + NIR laser had a maturation ratio of 16.0 %, which is significantly less than those of UCMS@Pep-RB and UCMS@Pep-RB + NIR groups. The percentage of mature DCs increases from 14.3 to 28.2% after treatment with UCMS@Pep, suggesting that AL-9 peptide could promote DCs maturation. Combined with NIR laser, UCMS@Pep-RB could stimulate the LLC cells to release more DAMPs. The UCMS@Pep-RB + NIR laser group had 50.4 % mature DCs, which is almost thrice that of the control group and NIR laser group. These results indicate that UCMS@Pep alone could promote DCs maturation. Combined with PDT, UCMS@Pep-RB could trigger strong DCs maturation in vitro owing to the upregulated expression of DAMPs in the tumor cells.

DCs maturation could further release cytokines that control immune cells, such as TNF-α and IL-12. Cytokines could bridge different immune cells through a paracrine and autocrine fashion, thereby exerting an imperative role for innate and adaptive immunity. Moreover, cytokines can control proliferation, differentiation, effector functions, and survival of leukocytes, especially T cells [37, 38]. TNF-α, a cytokine associated with inflammation, has been identified to induce rapid hemorrhagic necrosis of cancer cells [39]. IL-12 mediates DCs maturation, activates T cells, and increases the level of cytotoxic cells, thereby inducing the apoptosis of tumor cells [40]. In our in vitro study, the UCMS@Pep and UCMS@Pep-RB + NIR laser groups have higher levels of TNF-α and IL-12 (Fig. 4G, H) than the control group. Moreover, the highest cytokine secretion levels are observed in the LLC cells treated with UCMS@Pep-RB + NIR laser.

### Inhibition of metastatic spine tumor with UCMS@Pep-aPDL1-RB

**Fig. 5:**
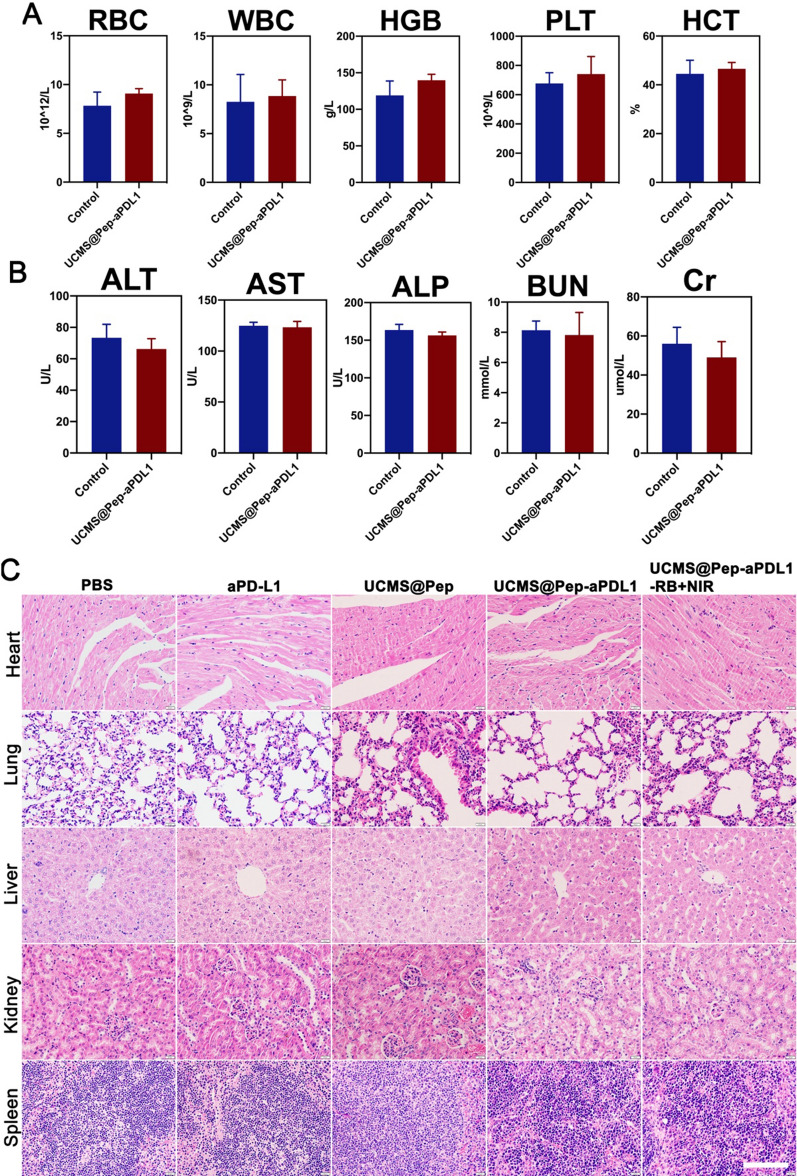
Biosafety in vivo of UCMS@Pep-aPDL1. **A** Results of routine blood count in mice treated with PBS and UCMS@Pep-aPDL1 (*n* ≥ 3); **B** Biochemistry indexes in mice treated with PBS and UCMS@Pep-aPDL1 (n ≥ 3); **C** H&E staining results obtained for the major organs of mice subjected to different treatments (Scale bar = 100 μm)

Based on the results of the in vitro experiments, we next performed in vivo experiments to confirm the amplified antitumor effect of UCMS@Pep and its synergistic effect with anti PD-L1 therapy using C57/BL6 mice with a metastatic spinal tumor. Owing to the large pore of UCMS, the PD-L1 antibody was further loaded onto the UCMS@Pep to form UCMS@Pep-aPDL1. After the intravenous injection, the half-life time of UCMS@Pep-aPDL1 in blood is calculated to be 0.96 h based on the blood circulation curve (Additional file 1: Fig. S10), which facilitates its target accumulation at tumor site via EPR effect. Further, we studied the biodistribution of UCMS@Pep-aPDL1 in the spinal tumor-bearing mice. The accumulation of Cyanine7 (Cy7)-labeled UCMS@Pep-aPDL1 in the tumor tissue and major organs at 1, 4, 12, and 24 h was evaluated using in vivo imaging system (IVIS). There were no obvious abnormal behaviors observed in the mice after the systemic administration of UCMS@Pep-aPDL1. As illustrated in Additional file 1: Fig. S11, the retention quantity at the tumor site gradually increases, reaching the maximum values at 12 h after the injection and is still observed after 24 h. This result indicates the excellent accumulation of UCMS@Pep-aPDL1 in the tumors, which is a precondition for immune response activation. For all time points, accumulation of Cy7-labeled UCMS@Pep-aPDL1 in the main organs of the mice was also detected. The in vivo safety of UCMS@Pep-aPDL1 was further investigated by blood biochemistry tests and hematoxylin and eosin (H&E) staining of the main organs (Fig. 5 and Additional file 1: Fig. S12). The results suggest that there is no obvious hepatotoxicity or nephrotoxicity induced by UCMS@Pep-aPDL1. Furthermore, apoptosis and pathological changes in the major organs are not observed after treatment with UCMS@Pep-aPDL1 injections. These results suggest that UCMS@Pep-aPDL1 has favorable vivo biocompatibility.

**Fig. 6:**
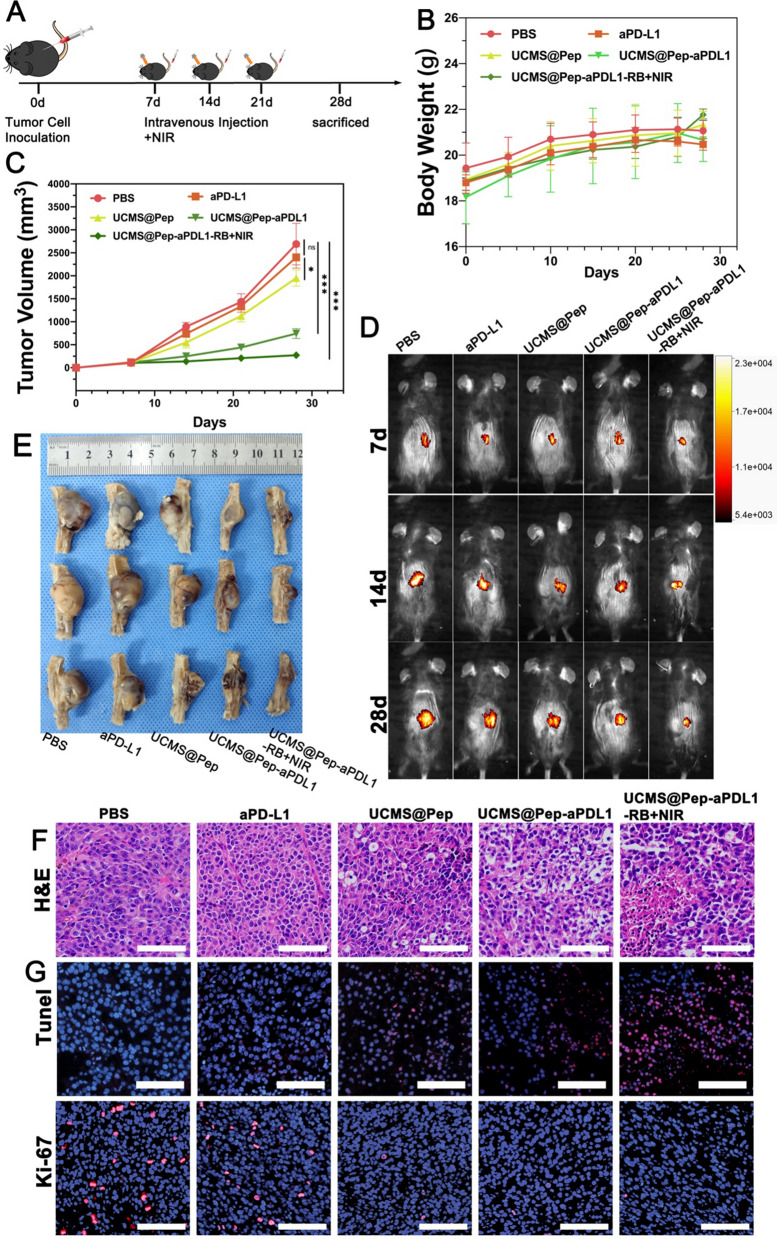
In vivo amplified anti-tumor effect of UCMS@Pep-aPDL1. **A** Schematics of the program used for spine metastasis tumor mode and IDO-based peptide tumor vaccine-mediated synergetic cancer treatment. Time-dependent (**B**) body weight and (**C**) tumor volume of mice in different groups. **D** Tumor progression in different groups evaluated by IVIS. **E** Tumor specimens extracted after the mice had been sacrificed. **F** H&E-stained histological images, **G** TUNEL-stained pathological changes and Ki-67-stained cellular proliferation in tumor tissues (scale bars = 100 μm). (*P < 0.05, **P < 0.01, ***P < 0.001, *n* > 3)

Sequentially, the antitumor effects of UCMS@Pep-aPDL1 were evaluated. Figure 6A presents a schematic of the in vivo therapy. After 7, 14, and 21 d, the mice were subjected to different interventions. At day 28, the mice were euthanized to harvest peripheral blood and tumor tissues. The body weights of the mice were documented every 5 d. There was no significant difference noted between the groups except for the control group (Fig. 6B). The tumor progression was evaluated by IVIS. Figure 6 C and D reveal the tumor progression with PBS treatment. The free anti-PDL1 group exhibited an undesirable antitumor effect that is ascribed to its poor accumulation in the tumor tissue and relatively low systematic administration doses. A moderate increase in the tumor volume was noted for the UCMS@Pep group. For the UCMS@Pep-aPDL1 group, a slight increase in the tumor size was observed at the last intervention, while there was no tumor progression for the UCMS@Pep-aPDL1-RB + NIR laser group. Meanwhile, the progression of the tumors treated with UCMS@Pep and UCMS@Pep-aPDL1 was evidently slower than that of the anti-PDL1 blockade alone. The results demonstrate that antitumor efficiency could be enhanced by combining peptide vaccines and PDT. At the end of the treatments, the mice were euthanized and their spines with tumors were harvested. The tumor was weighed and the results are shown in Additional file 1: Fig. S13. UCMS@Pep-aPDL1-RB plus NIR laser had the smallest tumor size, followed by UCMS@Pep-aPDL1 (Fig. 6E). The H&E staining of the tumor tissue showed that UCMS@Pep-aPDL1 and UCMS@Pep-aPDL1-RB + NIR laser underwent serious nuclei dissolution and disappeared cell morphology compared with the PBS and anti-PD-L1 monotherapy treatments (Fig. 6F). UCMS@Pep also displays moderate apoptosis or necrosis compared to PBS with intact nuclei. Transferase-mediated d-UTP nick-end labeling and proliferating cell nuclear antigen Ki-67 as the indices to evaluate the necrosis and proliferation capacity, respectively, were also evaluated by immunofluorescence. UCMS@Pep-aPDL1 and UCMS@Pep-aPDL1-RB + NIR laser treatments resulted in clearly more tumor cell apoptosis and fewer cells in division than the anti PD-L1 treatment.

Therefore, UCMS@Pep-aPDL1-RB combined with PDT was shown to have the best therapeutic efficacy against tumor progression, followed by UCMS@Pep-aPDL1 and then UCMS@Pep therapy. These results indicate that the integration of AL-9 peptide vaccine with PDT has a synergistic effect in promoting antitumor effect with anti PD-L1 therapy.

### Immunologic response and accumulation of DCs and cytotoxic CD8^+^ T cell with in vivo UCMS@Pep-aPDL1-RB

The in vitro experiments showed that the photodynamic effect of UCMS@Pep-RB under NIR laser irradiation can trigger the ICD of tumor cells and further activate DCs. The antitumor effect of UCMS@Pep-aPDL1-RB in vivo also indicates that the IDO-derived peptide vaccine has an important role in the tumor microenvironment. We analyzed the tumor microenvironment to further explore the mechanism of the enhanced immune response with UCMS@Pep-aPDL1-RB in vivo.

**Fig. 7:**
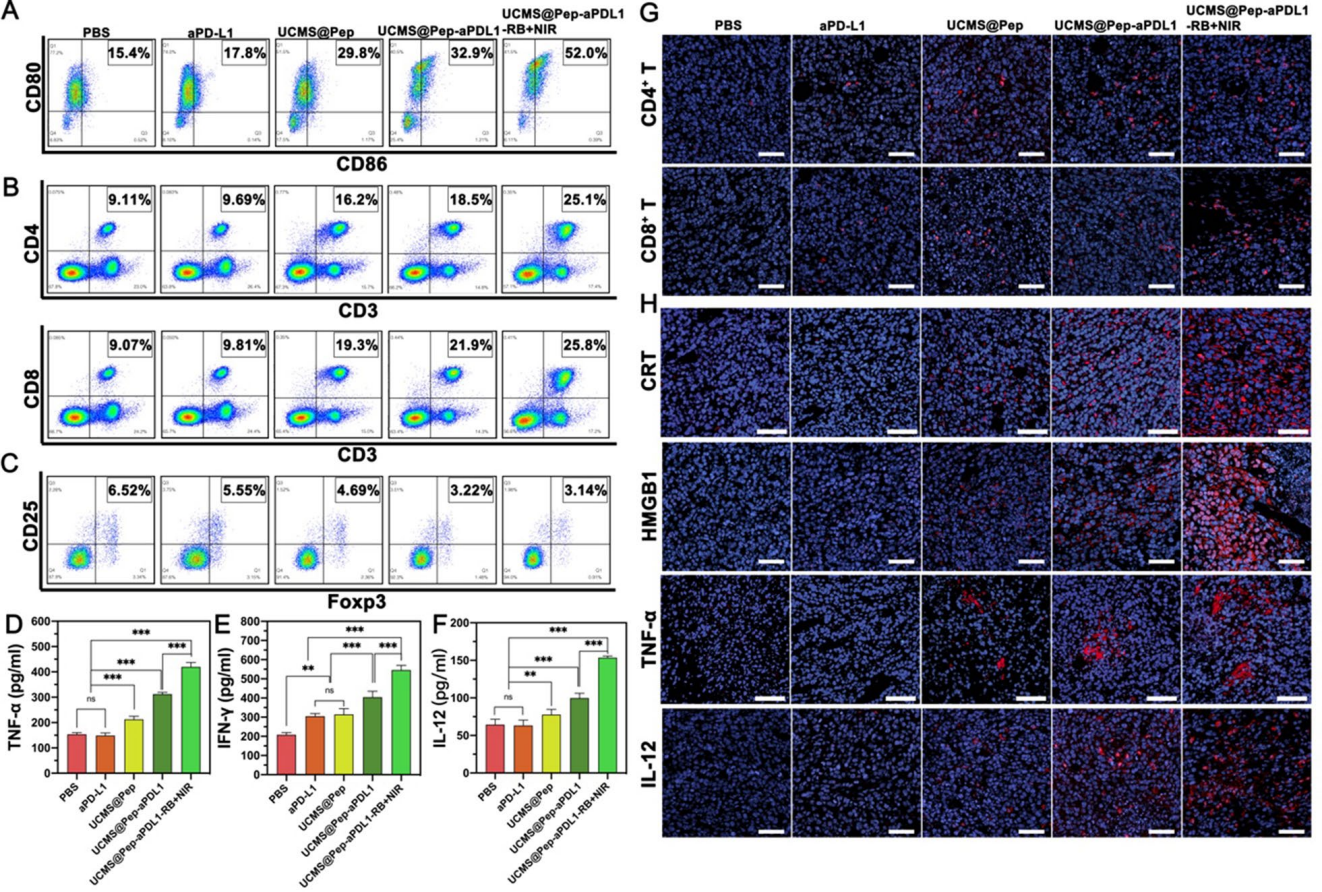
In vivo immune system enhancement by UCMS@Pep-aPDL1. **A**–**C** Flow cytometric analyses of DCs, CD4 and CD8 T cells and Treg cells in the tumor tissues of mice immunized using different interventions. Secretion levels of (**D**) TNF-α, **E** IFN-γ, and **F** IL-12 in the serum of peripheral blood from treated mice. **G** Infiltration of CD4 and CD8 T cells in tumor sites detected by CLSM (scale bar = 50 μm). **H** Expression of CRT, HMGB1, and the infiltration of cytokines TNF-α and IL-12 in tumor sites evaluated by CLSM (scale bar = 50 μm). (*P < 0.05, **P < 0.01, ***P < 0.001, *n* > 3)

First, the DCs maturation and infiltration of T cells induced by UCMS@Pep-aPDL1-RB were investigated. The accumulation of mature DCs (CD11c^+^CD80^+^CD86^+^), CD4^+^ T cells (CD3^+^CD4^+^CD8^−^), CD8^+^ T cells (CD3^+^CD4^−^CD8^+^), and regulatory T cells (Treg; CD3^+^CD4^+^CD25^+^Foxp3^+^) in the tumor tissue were detected by flow cytometry (Fig. 7 A–C and Additional file 1: Figs. S14, S15). As shown in Fig. 7A, 15.4 and 17.8% of mature DCs in the PBS and anti-PDL1 antibody group, respectively, were present, while that in the UCMS@Pep group is 29.8 %, which is approximately 1.93 times higher than that in the PBS group. In addition, the UCMS@Pep-aPDL1 group has higher DCs maturation than the anti-PDL1 group. UCMS@Pep-aPDL1-RB + NIR laser was found to be the most efficient treatment in activating DCs, which was 3.37 times higher than that of the PBS group and significantly higher than the anti PD-L1 blockade group. The results illustrate that the reorganization of IDO-derived peptides and DAMPs leads to DCs maturation. Mature DCs typically present antigens to the T cells, and secrete cytokines to promote the activation and proliferation of T cells. The activated T cells could recognize and kill tumor cells with specific antigens [36]. In Fig. 7B, compared to the PBS group, the percentages of cytotoxic CD8^+^ T cells in UCMS@Pep-aPDL1 and UCMS@Pep-aPDL1-RB + NIR laser groups are 2.41 and 2.84 times higher, respectively, while those of the CD4^+^ T cells are 2.03 and 2.75 times higher, respectively. The results of the CD4^+^ T and CD8^+^ T cell infiltration from immunofluorescence exhibit a similar trend (Fig. 7G) as well.

In addition to DCs, CD4^+^, and CD8^+^ T cells, Tregs are essential immune cells that can inhibit the function of effective T cells and induce tumor cell immune escape in a tumor microenvironment [41]. In turn, the tumor cells can promote the formation of Tregs by the direct secretion of cytokines [42, 43]. Therefore, we also analyzed the Treg level in the tumor tissues. As shown in Fig. 7C, there are less Tregs in the UCMS@Pep, UCMS@Pep-aPDL1, and UCMS@Pep-aPDL1-RB + NIR laser groups as compared to the PBS and anti PD-L1 blockade groups, demonstrating that UCMS@Pep-aPDL1-RB with PDT could reverse an immunosuppressive microenvironment. Meanwhile, the infiltration and activation of immune cells in spleen is also an important observation of immune system. we also performed flow cytometric analysis of immune cells in mouse spleen and got similar results (Additional file 1: Fig. S16).

Additionally, the application of IDO-derived peptide vaccine was reported to inhibit the activity of IDO enzyme, which is a rate-limiting enzyme for the conversion of tryptophan (Trp) to kynurenine (Kyn) [44]. IDO can consume Trp that is necessary for T cell activation and proliferation, while Kyn aids the transformation of immature CD4^+^ T cells into suppressor T cells and thus inhibits the body’s immune function [45, 46]. Thus, we tested the Trp and Kyn levels in the blood serum and tumor tissues. As illustrated in Additional file 1: Fig. S17, the Kyn/Trp ratio in the UCMS@Pep group is less than that in the PBS group, indicating the lower IDO activity with the treatment of our nano-system. Hence, compared to anti PD-L1 or PBS intervention alone, UCMS@Pep decreases the level of IDO activity.

As mentioned in Sect. "Expression of ICD markers and DCs maturation for enhanced ICD for in vitro UCMS@Pep with NIR laser", when the immune system is activated, immune cells secrete cytokines, including TNF-α, IFN-γ, and IL-12. In cancer therapy, IFN-γ has a function similar to that of IL-12 in mediating DC maturation and activating T cells [47]. Therefore, the cytokines in the tumor microenvironment were analyzed in vivo. The immunofluorescence and ELISA assay results of the intratumor cytokines associated with the immunoresponse in Fig. 7D–F demonstrate that UCMS@Pep-aPDL1-RB + NIR laser induces a greater release of cytokines, consistent with the results of the in vitro study. Moreover, in the UCMS@Pep-aPDL1-RB + NIR laser group, the TNF-α level was approximately three times higher than that in the PBS group. IFN-γ and IL-12, which play an important role in T cell activation, were enhanced by 1.66 and 2.00 times than that of the PBS group.

In addition, CRT and HMGB1 exposure is necessary in increasing immunogenic killing, thereby acting as a representative biomarker to determine ICD. Therefore, the expression of CRT and HMGB1 in the tumor tissue was detected by immunofluorescence. As shown in Fig. 7H, PBS and anti PD-L1 blockade groups did not trigger CRT and HMGB1 exposure. Meanwhile, UCMS@Pep-aPDL1-RB with PDT exhibited the brightest fluorescence, suggesting the obvious increase in CRT and HMGB1, and subsequently, a potent ICD-inducing effect in vivo.

## Conclusions

In this study, we have successfully developed mesoporous silica nanoparticles modified with an IDO-derived peptide, PD-L1 blockade, and photosensitizer that could efficiently accumulate at the tumor site with prolonged retention time. UCMS@Pep-aPDL1 loaded with peptide vaccine and anti-PD-L1 antibody could significantly enhance the immune system compared to anti PD-L1 blockade alone. Moreover, the highest antitumor immune responses are observed with the combination therapy of UCMS@Pep-aPDL1-RB with PDT in the metastatic spinal tumor models in vivo. Our approach could provide a generalized nanoplatform to deliver a tumor-associated antigen-derived peptide vaccine with PDT to boost antitumor immunity and synergize ICB for cancer immunotherapy.

## Supplementary Information

**Additional file 1: Fig. S1.** Scanning electron microscopy image of as-prepared UCMS. **Fig. S2.** Purity of IDO peptide AL-9 determined by HPLC. **Fig. S3.** Mass spectrometric analysis of IDO peptide AL-9. **Fig. S4.** Fourier transform infrared spectra of as-prepared UCMS, UCMS@Pep, and UCMS@Pep-aPDL1. **Fig. S5.** (A) The fluorescent emission and (B) intensity value of FITC-labeled aPDL1 solution at different concentrations. **Fig. S6.** CLSM images of tumor cells treated with the complete culture medium and UCMS@Pep-RB for 4 h. The green fluorescence was emitted by UCMS@Pep-RB under irradiation with a 980 nm laser. **Fig. S7.** The cumulative release of RB from UCMS@Pep-aPDL1 under pH 7.4 and 5.5 at different time points. **Fig. S8.** Mean fluorescence intensity (MFI) of CRT expression in vitro (ns: not significant, *P < 0.05, **P < 0.01, ***P < 0.001, n=3) **Fig. S9.** MFI of HMGB1 expression in vitro (ns: not significant, *P < 0.05, **P < 0.01, ***P < 0.001, n=3)). **Fig. S10.** The blood circulation of UCMS@Pep-aPDL1 in mice after intravenous injection as determined by measuring Fe element at different time intervals (n=3). **Fig. S11.** Accumulation of UCMS@Pep-aPDL1 labeled by Cy7 in major organs and tumors and the related MFIs (n ≥ 3). **Fig. S12.** Results of routine blood count in mice treated with PBS and UCMS@Pep-aPDL1 (n ≥ 3). **Fig. S13.** Weight of tumors in every group. (*P < 0.05, **P < 0.01, ***P < 0.001, n≥3). **Fig. S14.** Overview of gating strategy for DCs (A) and Gating strategy of DCs (A) and T cells including CD4^+^ and CD8^+^ T cells and regular T cells (B). For DCs, briefly, firstly gating FSC-A versus FSC-H for single cells, followed by Live cells based on Zombie UV-negative cells (A, a-b). From viable cells, DCs (CD45^+^CD11c^+^) can be determined (A, c-e). Within the CD11c^+^ DCs, the maturation of DCs (CD80^+^CD86^+^) can be determined. For T cells, briefly, viable cells were determined by firstly gating FSC-A versus FSC-H for single cells, followed by lymphocytes based on FSC-A and SSC-A, and lastly CD45^+^ and Zombie UV-negative cells (B, a-c). From lymphocytes, CD4^+^ T cells (CD3^+^CD4^+^CD8^−^), CD8^+^ T cells (CD3^+^CD4^-^CD8^+^) can be determined (B, d-f). Within the CD4^+^ T cells, regulatory T cells can be determined as CD4^+^CD25^+^. **Fig. S15.** The quantitative results of DCs (A), CD4^+^ and CD8^+^ T cells (B,C) and Treg (D) filtration in tumor tissue (*P < 0.05, **P < 0.01, ***P < 0.001, n≥3). **Fig. S16.** Flow cytometric analyses of DCs, CD4 and CD8 T cells and Treg cells in the spleens of mice immunized using different interventions. **Fig. S17.** Kyn/Trp ratio in blood serum and tumor tissue. (*P < 0.05, **P < 0.01, ***P < 0.001, n≥3).
