# Supplementary material for: Peptide vaccine-conjugated mesoporous carriers synergize with immunogenic cell death and PD-L1 blockade for amplified immunotherapy of metastatic spinal
Source: J Nanobiotechnology. 2021 Aug 12;19:243. doi: 10.1186/s12951-021-00975-5 (PMC8362242; doi:10.1186/s12951-021-00975-5)
Supplement: Supplementary file 1 — Additional file 1: Fig. S1. Scanning electron microscopy image of as-prepared UCMS. Fig. S2. Purity of IDO peptide AL-9 determined by HPLC. Fig. S3. Mass spectrometric analysis of IDO peptide AL-9. Fig. S4. Fourier transform infrared spectra of as-prepared UCMS, UCMS@Pep, and UCMS@Pep-aPDL1. Fig. S5. (A) The fluorescent emission and (B) intensity value of FITC-labeled aPDL1 solution at different concentrations. Fig. S6. CLSM images of tumor cells treated with the complete culture medium and UCMS@Pep-RB for 4 h. The green fluorescence was emitted by UCMS@Pep-RB under irradiation with a 980 nm laser. Fig. S7. The cumulative release of RB from UCMS@Pep-aPDL1 under pH 7.4 and 5.5 at different time points. Fig. S8. Mean fluorescence intensity (MFI) of CRT expression in vitro (ns: not significant, *P < 0.05, **P < 0.01, ***P < 0.001, n=3) Fig. S9. MFI of HMGB1 expression in vitro (ns: not significant, *P < 0.05, **P < 0.01, ***P < 0.001, n=3)). Fig. S10. The blood circulation of UCMS@Pep-aPDL1 in mice after intravenous injection as determined by measuring Fe element at different time intervals (n=3). Fig. S11. Accumulation of UCMS@Pep-aPDL1 labeled by Cy7 in major organs and tumors and the related MFIs (n ≥ 3). Fig. S12. Results of routine blood count in mice treated with PBS and UCMS@Pep-aPDL1 (n ≥ 3). Fig. S13. Weight of tumors in every group. (*P < 0.05, **P < 0.01, ***P < 0.001, n≥3). Fig. S14. Overview of gating strategy for DCs (A) and Gating strategy of DCs (A) and T cells including CD4+ and CD8+ T cells and regular T cells (B). For DCs, briefly, firstly gating FSC-A versus FSC-H for single cells, followed by Live cells based on Zombie UV-negative cells (A, a-b). From viable cells, DCs (CD45+CD11c+) can be determined (A, c-e). Within the CD11c+ DCs, the maturation of DCs (CD80+CD86+) can be determined. For T cells, briefly, viable cells were determined by firstly gating FSC-A versus FSC-H for single cells, followed by lymphocytes based on FSC-A and SSC-A, and lastl [file 12951_2021_975_MOESM1_ESM.docx]

**Supplementary information**

**Peptide vaccine-conjugated mesoporous carriers synergize with immunogenic**

**cell death and PD-L1 blockade for amplified immunotherapy of metastatic spinal**

**tumor**

Zhenqing Wang^a,b,1^, Liang Chen^c,1^, Yiqun Ma^a,b^, Xilei Li^a,b^, Annan Hu^a,b^, Huiren Wang^a,b^, Wenxing Wang^c^, Xiaomin Li^c,*^, Bo Tian^a,b,*^, Jian Dong^a,b,d,*^

^a^ Department of Orthopaedic Surgery, Zhongshan Hospital, Fudan University, Shanghai， 200032, PR China.

^b^ Cancer Center, Zhongshan Hospital, Fudan University, Shanghai, 200032, PR China

^c^ Materdicine Lab, School of Life Sciences, Shanghai University, Shanghai, 200444, PR China.

^d^ Department of Orthopaedic Surgery, Shanghai Baoshan District Wusong Center Hospital, Zhongshan Hospital Wusong Branch, Fudan University, Shanghai, 200940, PR China.

^1^ These authors contributed equally to this work.

^*^ Corresponding authors E-mail addresses: [dong.jian@zs-hospital.sh.cn](mailto:dong-jian@zs-hospital.sh.cn) (J. Dong), [poetian@163.com](mailto:poetian@163.com) (B. Tian) [lixm@fudan.edu.cn](mailto:lixm@fudan.edu.cn) (X-M. Li).

**Methods**

**1.1 Materials**

1.1.1 Immunofluorescence:

Primary antibody: Anti-Calreticulin antibody (Proteintech, China); Anti-HMGB1antibody (Abcam, USA); Phalloidin (Green, Solarbio, China); Anti-CD4 antibody (Abcam, USA); Anti-CD8α antibody (Abcam, USA); Anti-TNF-α antibody (Abcam, USA); Anti-IL-12 antibody (Solarbio, China); Second antibody: Goat anti-rabbit IgG Cy3, Goat anti-mouse IgG Cy3 (Abcam, USA)

1.1.2 Flow-cytometry antibody:

DCs: CD11c-APC, CD80-PE, CD86-FITC, CD45-BV421, Live-APC-Cy7; T cells: Live-APC-Cy7, CD45-BV510, CD3-PE-Cy7, CD4-BV786, CD8-Percp-CY5.5, CD25-APC, Foxp3-BV421, purchased from Biolegend, USA.

**1.2 Synthesis of UCNPs (NaYF_4_:Yb/Er@NaYF_4_)**

NaYF_4_:20 %Yb/2 %Er core were prepared by the thermolysis method. YCl (0.78 mmol), YbCl_3_ (0.2 mmol), ErCl_3_ (0.02 mmol), OA (4.0 mL) and ODE (15.0 mL) were mixed together and heated to 140°C under vacuum until a clear solution formed, after that, the solution was cooled down to room temperature. A solution of NaOH (2.5 mmol) and NH_4_F (4.0 mmol) in methanol (10 mL) was added and the resultant mixture was stirred for half an hour. The reaction mixture was then heated to 70°C and maintained for half an hour to remove the methanol. Afterward, the solution was heated to 300°C and maintained for 100 min under a gentle argon flow. Then, the solution was cool down to room temperature and the nanoparticle products were centrifuged and washed twice with ethanol. The nanoparticles were finally dispersed in 10 mL of cyclohexane for further use.

To coat an inert layer of NaYF_4_ on NaYF_4_:Yb/Er core, 2.5 mL of the purified NaGdF_4_: Yb/Er core nanoparticles solution (~ 0.25 mmol) were mixed with 4.0 mL of OA and 6.0 mL of ODE. The flask was pumped down at 70°C for 30 min to remove cyclohexane. After that, the system was switched to argon flow and the reaction mixture was further heated to 280°C at a rate of ~ 20 °C/min. Then pairs of Y-OA (0.10 M, 1.0 mL) and Na-TFA-OA (0.40 M, 0.50 mL) precursors were alternately introduced by dropwise addition at 280°C and the time interval between each injection was 15 min. Finally, the obtained NaYF_4_: Yb/Er@NaYF_4_ core@shell nanoparticles were precipitated and washed in the same way as the core nanoparticles, and dispersed in 5 mL cyclohexane.

**1.3 Characterizations of UCMS@Pep-aPDL1**

The nanoparticles were observed by transmission electron microscopy (TEM) under accelerated voltage of 200 kV (FEI, American). Scanning electron microscopy (SEM) images were captured using field emission scanning electron microscopy (FESEM, Hitachi S-4800, Japan). UV–vis–NIR absorption spectra were measured on a Shimadz spectrophotometer (UV-3150) (Japan). Size distribution and Zeta potential of the samples were recorded by using Zetasizer Nano ZS apparatus (Malvern, UK). Fourier transform infrared (FTIR) spectra were recorded using a NICOLET MX-1E FTIR spectrometer.

**1.4 Intracellular uptake in vitro**

LLC cells were seeded into a glass-bottom culture dish (Nest, China) at a concentration of 2×10^4^ cells per well and cultured for 24 h for fully attachment. The UCMS@Pep-aPDL1 (50 μg/mL) were added to the dish and incubated for 6 h. Then, cells were fixed using 4% paraformaldehyde, and then cell nucleus was stained with DAPI for the confocal laser scanning microscope (CLSM) (Olympus FV3000, Japan) visualization. The upconverting luminescence (UCL) of UCNPs was visualized for localization of the nanocarriers.

**1.5 Biodistribution of UCMS@Pep-aPDL1 in mice**

LLC spine metastasis model C57BL/6 mice were injected with Cy7-labeled UCMS@Pep-aPDL1 (100 μL) intravenously. Then the mice were euthanized at different time points (1h, 6h, 12h, 24h) and major organs (heart, liver, spleen, lung, kidney), spine with tumor were obtained. The fluorescence of UCMS@Pep-aPDL1 accumulation in major organs were detected and the intensity was calculated via in vivo imaging system.

**1.6 In vivo half-life time of UCMS@Pep-aPDL1**

The blood circulation of UCMS@Pep-aPDL1 was assessed by monitoring the Si concentration in the blood. Briefly, UCMS@Pep-aPDL1 dispersed in PBS was intravenously injected into mice. The blood samples were taken from the eyeballs of the mice at different time points post injection, and then digested in aqua regia for inductively coupled plasma-atomic emission spectroscopy (ICP-AES) measurement.

**1.7 In vivo toxicity analysis of UCMS@Pep-aPDL1**

Routine blood cell tests, blood biochemistry analysis, and H&E staining were carried out to evaluate the in vivo toxicology of UCMS@Pep-aPDL1. C57BL/6 female mice (6-8 weeks) were divided into two groups: UCMS@Pep-aPDL1 injection group and the control group (n = 6). Mice in UCMS@Pep-aPDL1 group were treated with 150 μL UCMS@Pep-aPDL1 intravenously, while the mice in the control group were injected with PBS. After last intervention, peripheral blood samples were collected from the mice by removing the eyeballs for the routine blood test and blood biochemical examinations including Alkaline phosphatase (ALP), Alanine aminotransferase (ALT), Aspartic transaminase (AST), Blood urine nitrogen (BUN), Creatinine (Cr). Then, the mice were sacrificed after euthanasia to dissect them and the major organs (heart, liver, spleen, lung, and kidney) were obtained and then fixed in 4% Paraformaldehyde and paraffin-embedded, followed with H&E staining. The H&E staining images were taken using an optical microscope.

**1.8 IDO activity evaluation in serum and tumor tissue**

To evaluate the IDO activity in vivo, the level of Kyn and Trp in blood serum and tumor tissue was detected via and the ratio of Kyn/Trp was performed[39]. Briefly, blood serum and tumor tissue of mice were harvest; then 50 μL of serum sample was added into 150 μL of methanol, vortexed for 1 min; then the mixture was centrifuged at 12000 rpm for 10 min, the supernatant were taken for analysis. For tumor tissue sample: a certain weight of sample was added into 1 mL pure methanol, added zirconia grinding beads and vortexed to mix, then the mixture was centrifuged at 13000 rpm at 4°C for 10 min; then filtered with 0.22 μm filter membrane. The above mixture was analyzed by X calibur 3.0 (Thermo, U.S.A.).

**Supporting Figures**


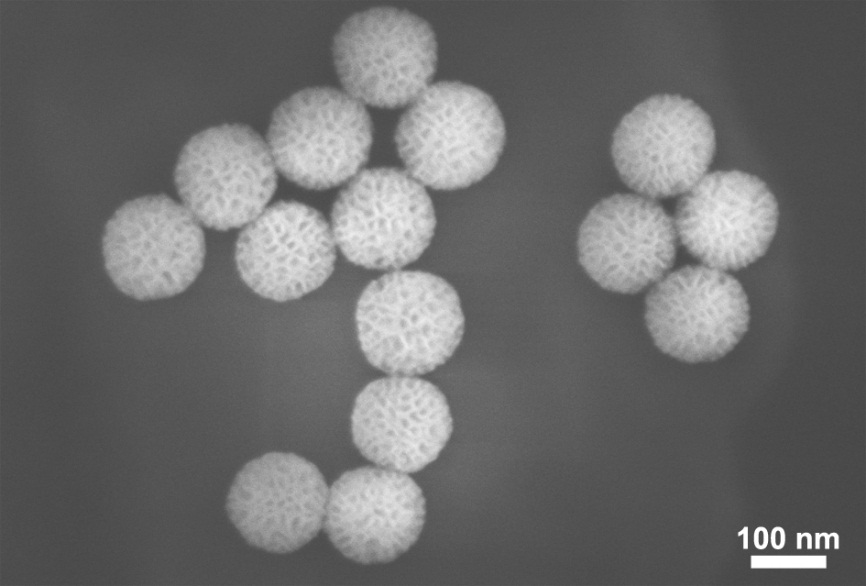


**Fig. S1.** Scanning electron microscopy image of as-prepared UCMS.


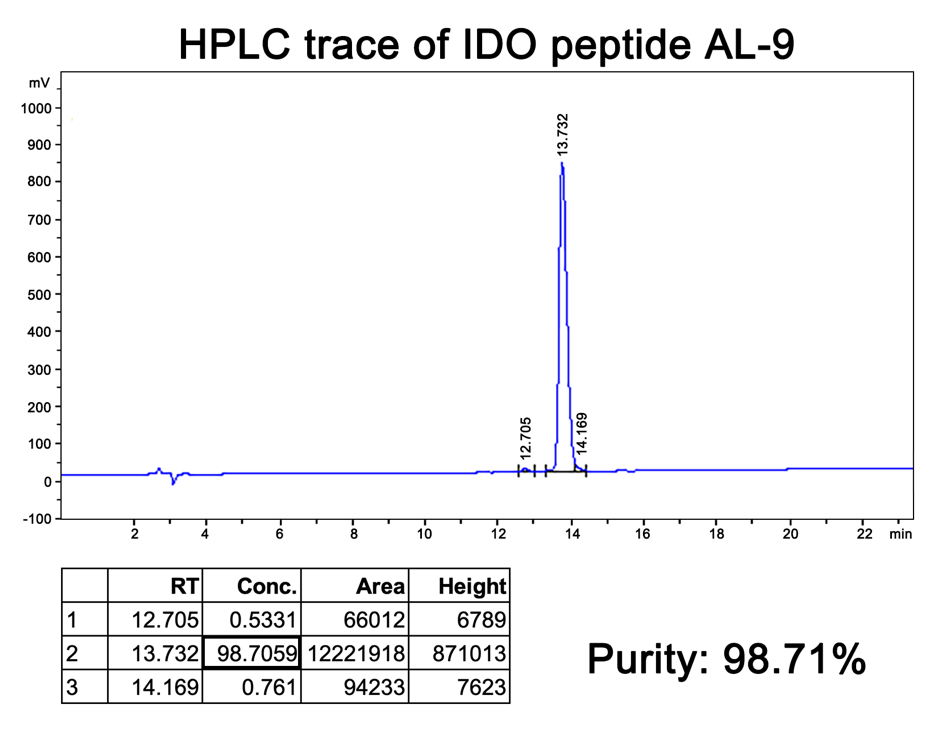


**Fig. S2.** Purity of IDO peptide AL-9 determined by HPLC.


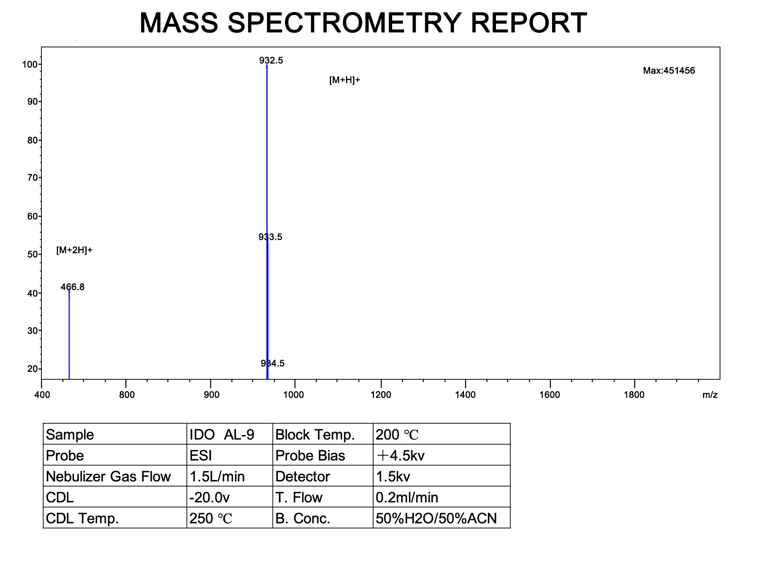


**Fig. S3.** Mass spectrometric analysis of IDO peptide AL-9.


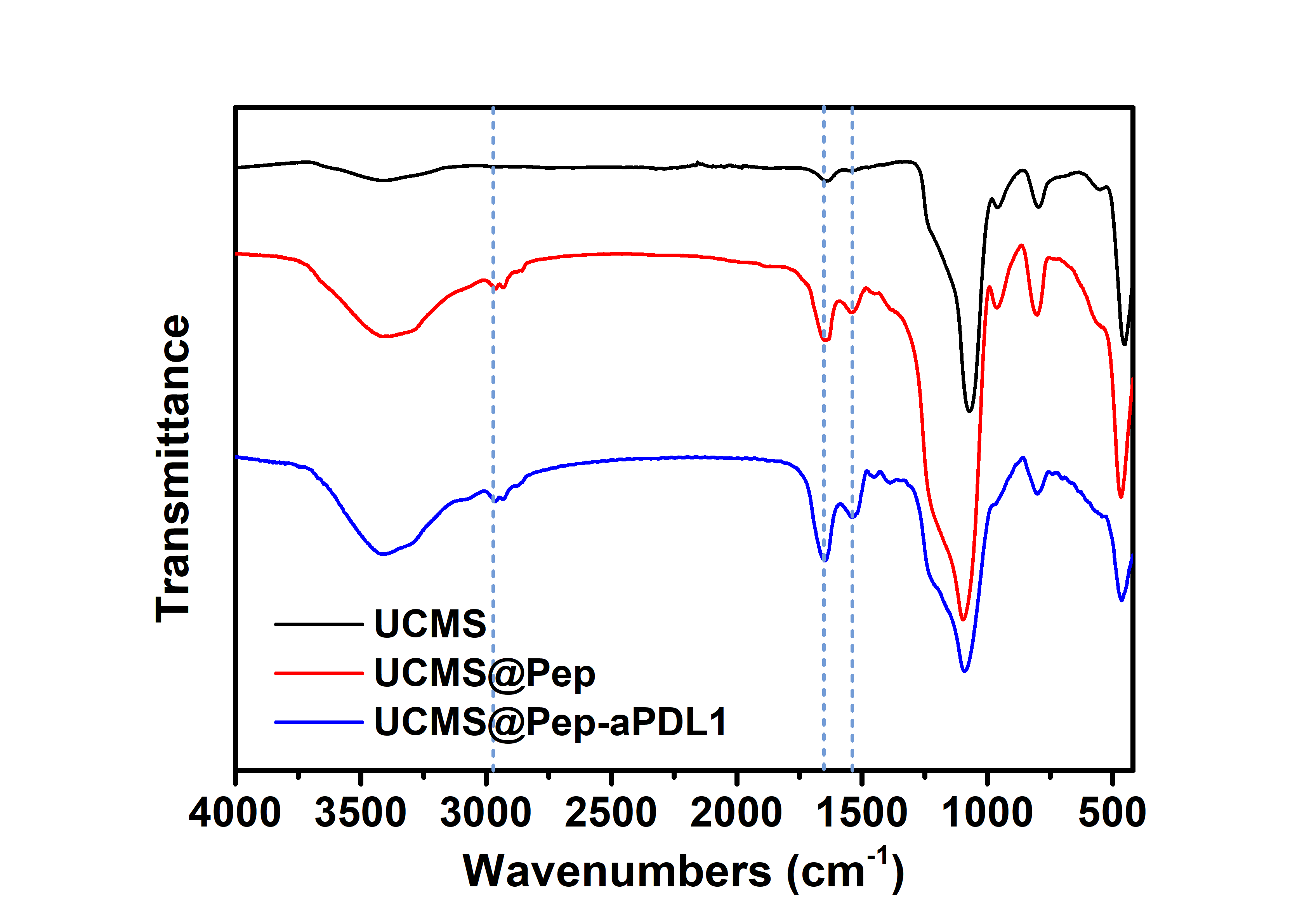


**Fig. S4.** Fourier transform infrared spectra of as-prepared UCMS, UCMS@Pep, and UCMS@Pep-aPDL1.


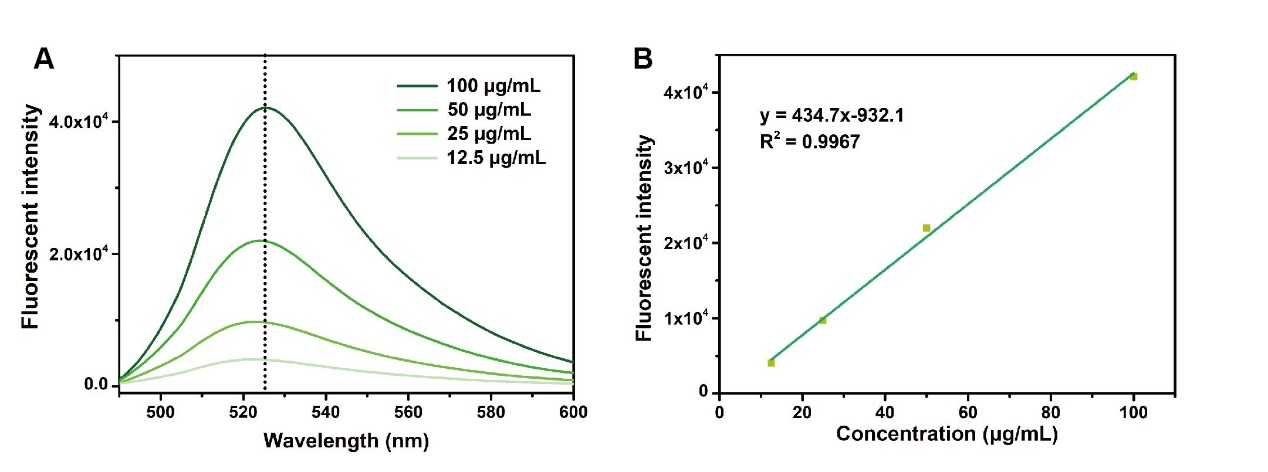


**Fig. S5.** (A) The fluorescent emission and (B) intensity value of FITC-labeled aPDL1 solution at different concentrations.


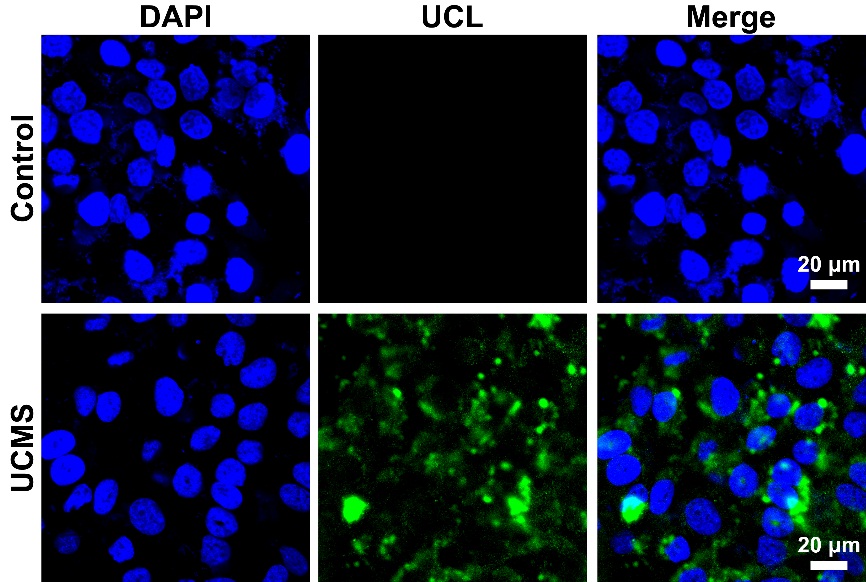


**Fig. S6.** CLSM images of tumor cells treated with the complete culture medium and UCMS@Pep-RB for 4 h. The green fluorescence was emitted by UCMS@Pep-RB under irradiation with a 980 nm laser.





**Fig. S7.** The cumulative release of RB from UCMS@Pep-aPDL1 under pH 7.4 and 5.5 at different time points.


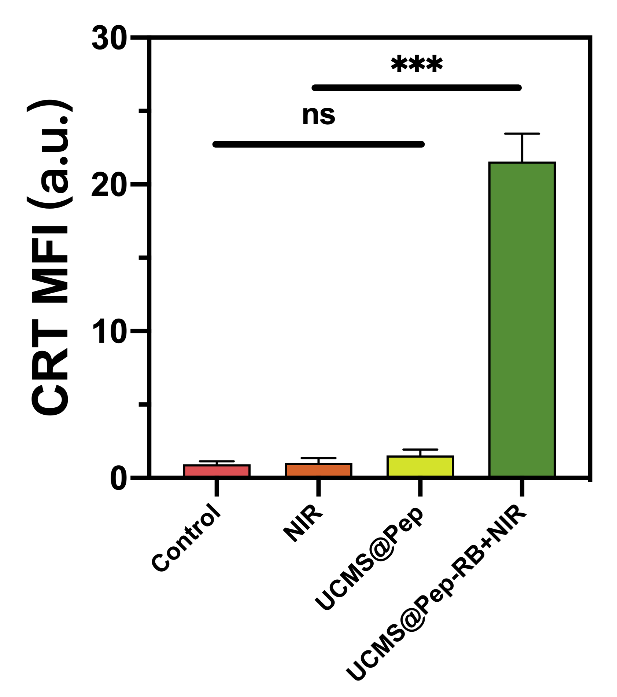


**Fig. S8.** Mean fluorescence intensity (MFI) of CRT expression in vitro (ns: not significant, **P* < 0.05, ***P* < 0.01, ****P* < 0.001, *n*=3)


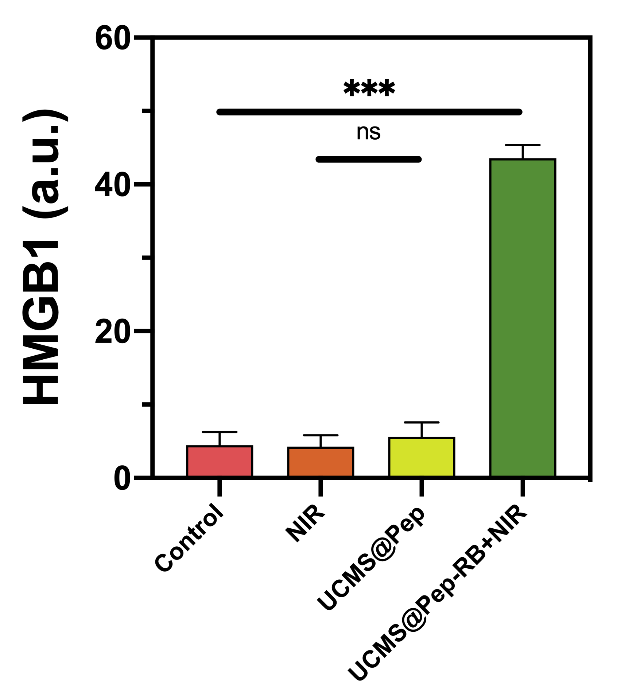


**Fig. S9.** MFI of HMGB1 expression in vitro (ns: not significant, **P* < 0.05, ***P* < 0.01, ****P* < 0.001, *n*=3))





**Fig. S10.** The blood circulation of UCMS@Pep-aPDL1 in mice after intravenous

injection as determined by measuring Fe element at different time intervals (n=3).


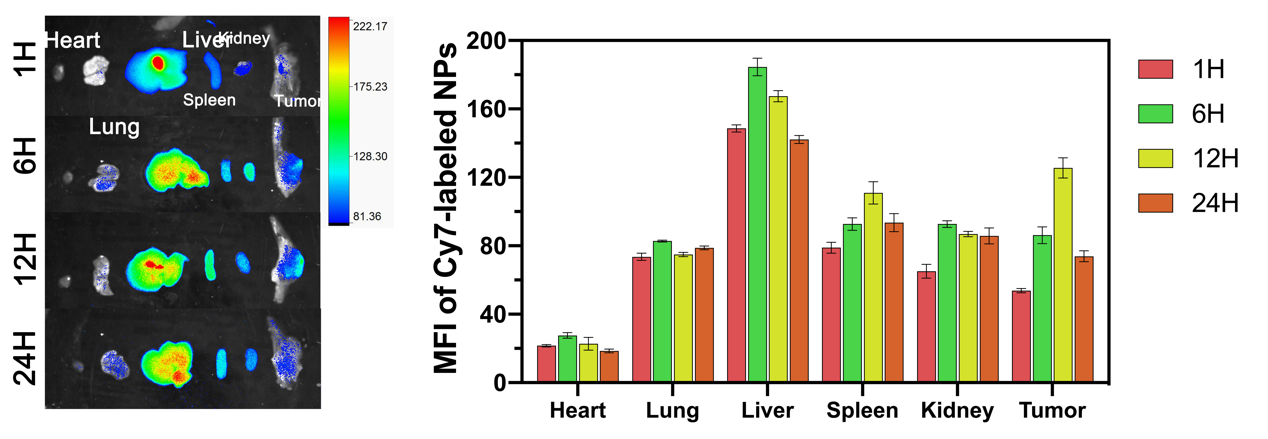


**Fig. S11.** Accumulation of UCMS@Pep-aPDL1 labeled by Cy7 in major organs and tumors and the related MFIs (*n* ≥ 3).


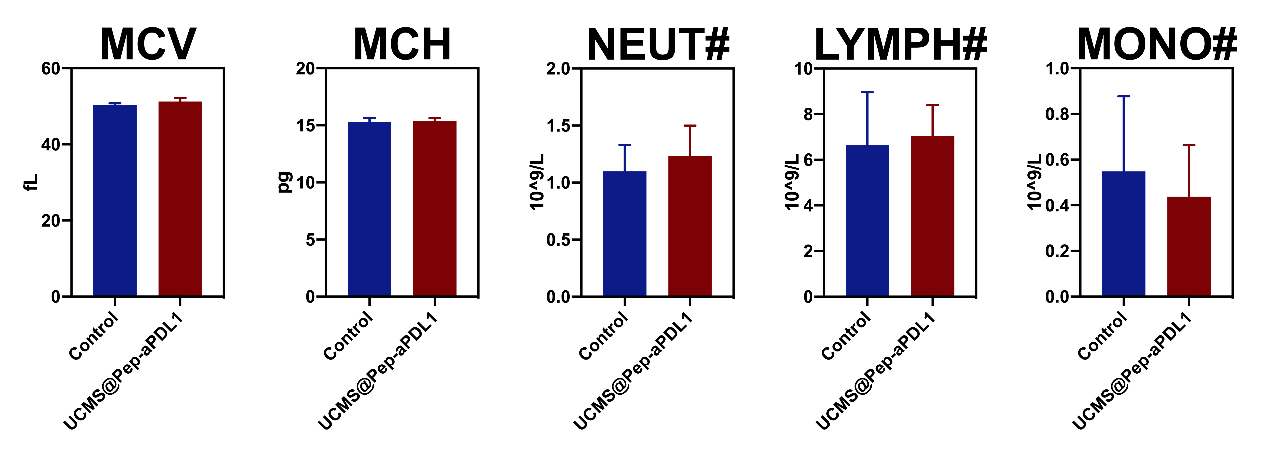


**Fig. S12.** Results of routine blood count in mice treated with PBS and UCMS@Pep-aPDL1 (*n* ≥ 3).


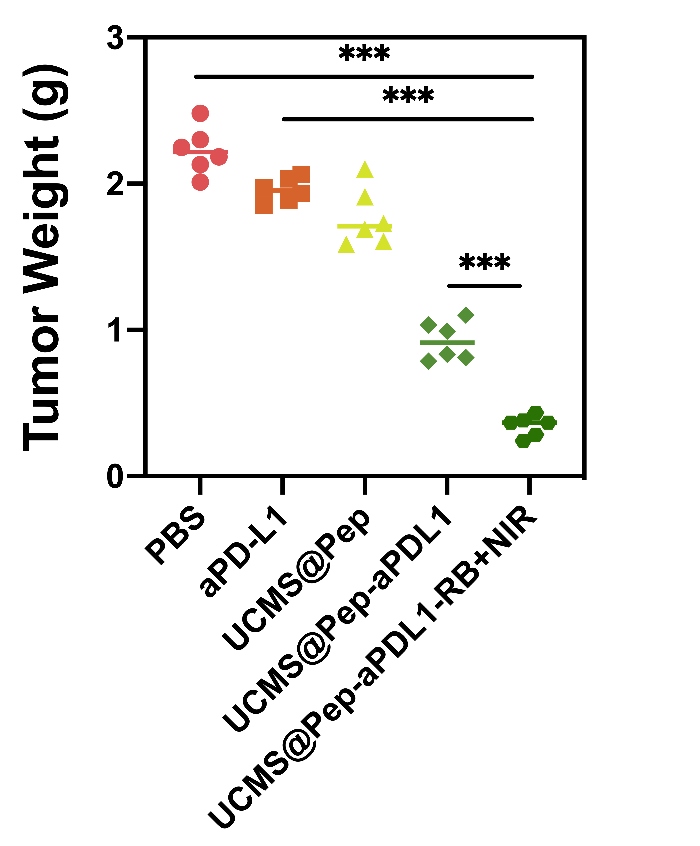


**Fig. S13.** Weight of tumors in every group. (**P* < 0.05, ***P* < 0.01, ****P* < 0.001, *n*≥3)


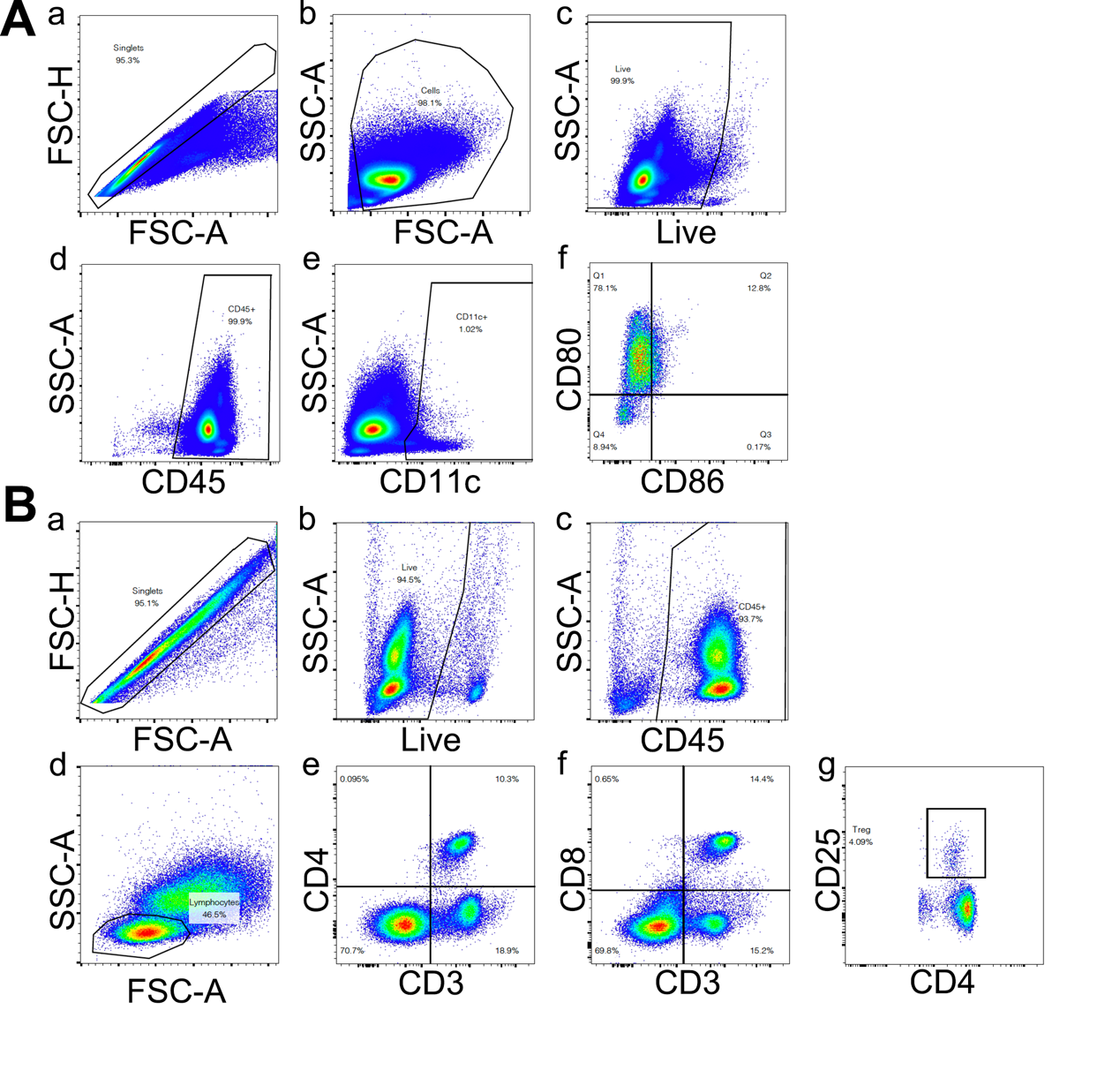


**Fig. S14.** Overview of gating strategy for DCs (A) and Gating strategy of DCs (A) and T cells including CD4^+^ and CD8+ T cells and regular T cells (B). For DCs, briefly, firstly gating FSC-A versus FSC-H for single cells, followed by Live cells based on Zombie UV-negative cells (A, a-b). From viable cells, DCs (CD45^+^CD11c^+^) can be determined (A, c-e). Within the CD11c^+^ DCs, the maturation of DCs (CD80^+^CD86^+^) can be determined. For T cells, briefly, viable cells were determined by firstly gating FSC-A versus FSC-H for single cells, followed by lymphocytes based on FSC-A and SSC-A, and lastly CD45^+^ and Zombie UV-negative cells (B, a-c). From lymphocytes, CD4^+^ T cells (CD3^+^CD4^+^CD8^-^), CD8^+^ T cells (CD3^+^CD4^-^CD8^+^) can be determined (B, d-f). Within the CD4^+^ T cells, regulatory T cells can be determined as CD4^+^CD25^+^.


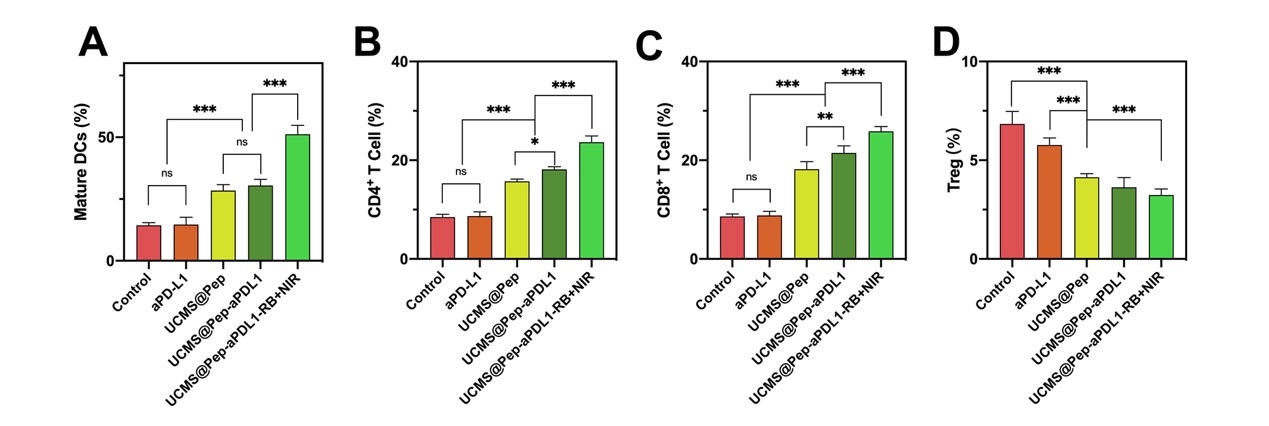


**Fig. S15.** The quantitative results of DCs (A), CD4^+^ and CD8^+^ T cells (B,C) and Treg (D) filtration in tumor tissue (**P* < 0.05, ***P* < 0.01, ****P* < 0.001, *n*≥3)


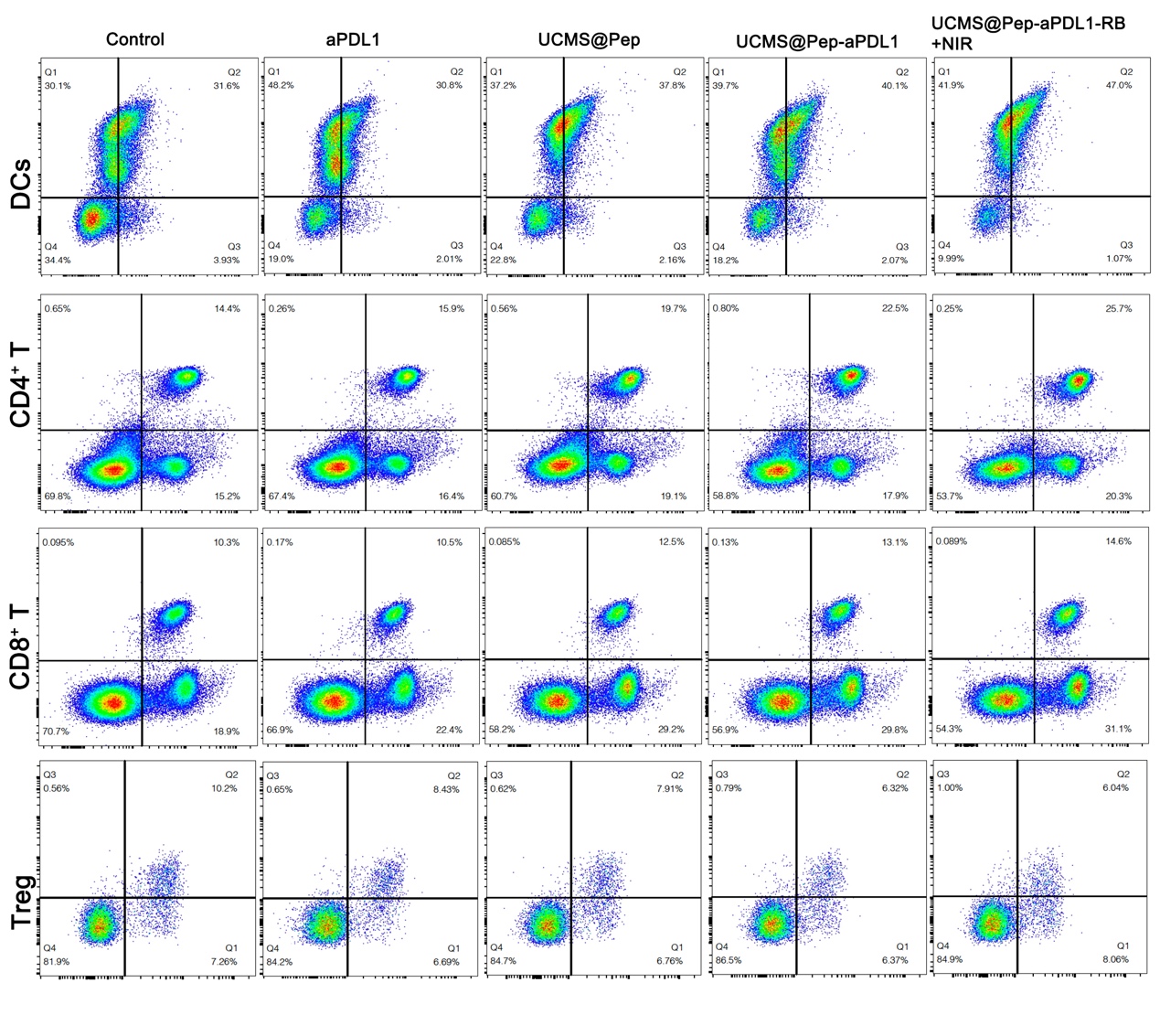


**Fig. S16.** Flow cytometric analyses of DCs, CD4 and CD8 T cells and Treg cells in the spleens of mice immunized using different interventions.


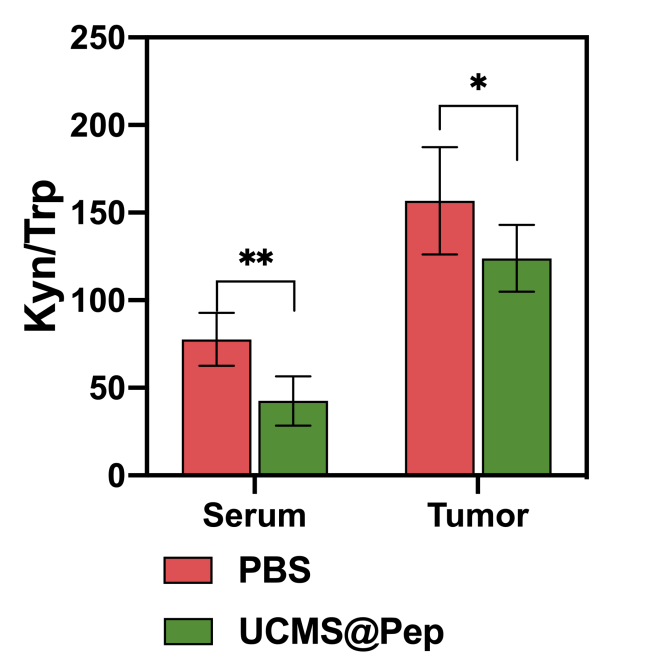


**Fig. S17.** Kyn/Trp ratio in blood serum and tumor tissue. (**P* < 0.05, ***P* < 0.01, ****P* < 0.001, *n*≥3)
